# Cytosolic co-delivery of miRNA-34a and docetaxel with core-shell nanocarriers via caveolae-mediated pathway for the treatment of metastatic breast cancer

**DOI:** 10.1038/srep46186

**Published:** 2017-04-06

**Authors:** Li Zhang, Xin Yang, Yaqi Lv, Xiaofei Xin, Chao Qin, Xiaopeng Han, Lei Yang, Wei He, Lifang Yin

**Affiliations:** 1 Department of Pharmaceutics, School of Pharmacy, China Pharmaceutical University, Nanjing 210009, P.R. China; 2 Jiangsu Key Laboratory of Druggability of Biopharmaceuticals, China Pharmaceutical University, Nanjing 210009, P.R. China

## Abstract

Co**-**delivery of microRNAs and chemotherapeutic drugs into tumor cells is an attractive strategy for synergetic breast cancer therapy due to their complementary mechanisms. In this work, a core-shell nanocarrier coated by cationic albumin was developed to simultaneously deliver miRNA-34a and docetaxel (DTX) into breast cancer cells for improved therapeutic effect. The co-delivery nanocarriers showed a spherical morphology with an average particle size of 183.9 nm, and they efficiently protected miRNA-34a from degradation by RNase and serum. Importantly, the nanocarriers entered the cytosol via a caveolae-mediated pathway without entrapment in endosomes/lysosomes, thus improving the utilization of the cargo. *In vitro,* the co-delivery nanocarriers suppressed the expression of anti-apoptosis gene Bcl-2 at both transcription and protein levels, inhibited tumor cell migration and efficiently induced cell apoptosis and cytotoxicity. *In vivo*, the co-delivery nanocarriers prolonged the blood circulation of DTX, enhanced tumor accumulation of the cargo and significantly inhibited tumor growth and metastasis in 4T1-tumor bearing mice models. Taken together, the present nanocarrier co-loading with DTX and miRNA-34a is a new nanoplatform for the combination of insoluble drugs and gene/protein drugs and provides a promising strategy for the treatment of metastatic breast cancer.

Breast cancer is the leading cause of cancer death in women worldwide, and metastasis-related recurrence accounts for over 90% of treatment failure clinically^1^,^2^. Chemotherapy, one of the three main approaches (parallel to radiotherapy and surgery) for cancer treatment, continues to play a critical role in clinical cancer treatment. However, conventional chemotherapy currently faces formidable challenges due to the occurrence of serious adverse effects and the development of multidrug resistance^3^,^4^. On the other hand, the RNA interference (RNAi) technique, an endogenous pathway that induces gene silencing effects at a post-translational level based on small interfering RNAs (siRNA) or microRNAs (miRNAs), has emerged as a novel and promising strategy for cancer therapy^5^,^6^. Moreover, miRNA-based therapy can increase the sensitization of cancer cells to conventional chemotherapeutic drugs by down-regulating efflux transporters, silencing anti-apoptotic genes, reverting the epithelial-to-mesenchymal transition (EMT), suppressing tumor angiogenesis, promoting cell apoptosis and autophagy^7^,^8^. Therefore, the combination of chemotherapy and RNAi is a potent strategy for synergistic cancer therapy through their different mechanisms^9^,^10^,^11^.

miRNA-34a is a potent down-regulated tumor suppressor, but it is defective in various human cancers such as breast, lung, kidney and pancreatic cancers. By targeting multiple genes, such as Bcl-2, cyclin D1 and silent information regulator 1, miRNA-34a is able to suppress tumor angiogenesis, induce cancer cell apoptosis, block proliferation, revert the EMT, and thereby inhibit tumor growth^12^,^13^,^14^. Docetaxel (DTX) is a broad-spectrum chemotherapeutic drug that possesses strong activity against many human cancers, such as metastatic breast, lung, prostate and ovarian cancers, through its binding to β-tubulin and stabilizing microtubules, thus leading to cell cycle arrest and cell apoptosis^15^. However, the clinical application of DTX is greatly limited by its poor water-solubility, rapid clearance rate, adverse side effects, poor tumor penetration and the occurrence of drug resistance^16^,^17^. In the marketed formulations, Taxotere^®^ and Duopafei^®^, high concentration of Tween 80 and ethanol has to be added to improve drug solubility, therefore resulting in adverse side effects including acute hypersensitivity, fluid retention, neurotoxicity and febrile neutropenia^18^.

Based on their complementary action mechanisms, we hypothesize that the combination of miRNA-34a and DTX may achieve synergistic therapy effects in breast cancer treatment. However, the development of carriers for the co-delivery of miRNAs and chemotherapeutic agents is a great challenge because of their drastically different physicochemical properties. In general, most chemotherapeutic compounds are poorly water-soluble, while miRNAs are polyanionic molecules with high water-solubility and high molecular weights. Moreover, systemic administration of naked miRNAs is greatly hindered by nuclease-labile degradation, rapid elimination, poor cellular uptake and lack of tissue selectivity^19^. To address these problems, a number of conventional nanocarriers based on cationic polymers such as poly(ethylenimine) (PEI) and dendrimers have been developed^20^,^21^. However, due to poor biocompatibility, low drug payload and complicated preparation, the clinical application of these nanocarriers is greatly limited. Moreover, these nanocarriers generally enter cells via endocytic pathways with entrapment in endosomes/lysosomes, thus leading to the degradation of drugs, especially gene drugs, and consequently compromising their therapeutic effects^22^,^23^. Accordingly, enhanced co-delivery performance of miRNAs and DTX into cytosol using nanocarriers is highly desirable.

We previously reported that a globular protein-coated nanoparticle obtained cellular entry without entrapment in endosomes/lysosomes via a caveolae-mediated pathway^24^,^25^. Encouraged by these findings, a core-shell nanocarrier coated by cationic bovine serum albumin (CBSA) was developed in the present study for the co-delivery of miRNA-34a and DTX (Fig. 1a). It was expected that the designed nanocarriers would achieve enhanced drug delivery performance via the caveolae-mediated pathway, highly accumulate at the tumor site via the enhanced permeation and retention (EPR) effect and prolonged circulation time, and finally achieve synergistic treatment of metastatic breast cancer through the combination of miRNA-34a and DTX (Fig. 1b). To obtain a proof of this concept, various experiments were performed, including characterization of the nanocarriers, cellular internalization, pharmacokinetics, biodistribution, antitumor efficacy, etc.

## Results

### Preparation and characterization of the core-shell nanocarriers

CBSA was obtained from BSA through modification of ethylenediamine by amide linkages in order to condense miRNA-34a (Supplementary Fig. S1). The IEF results showed that the pI of CBSA shifted to 8-9 from 4.6 (BSA), indicating the successful cationization of BSA (Supplementary Fig. S2).

The core-shell nanocarriers based on nanoemulsion-templates were prepared by a high-pressure homogenizing method. To confirm the core-shell structure of nanocarriers, microscale dual fluorescence-labeled carriers were prepared and observed by confocal laser scanning microscopy (CLSM). The cores were loaded with coumarin-6 (C6, green) and CBSA was labeled with rhodamine B isothiocyanate (RBITC, red). As shown in Fig. 2a, robust green fluorescence was seen within the core of the particles and bright red fluorescence was displayed in the rim around the core; yellow fluorescence around the cores was observed in the merged image, indicating the well-defined core-shell structure of the nanocarriers.

**Figure 1:**
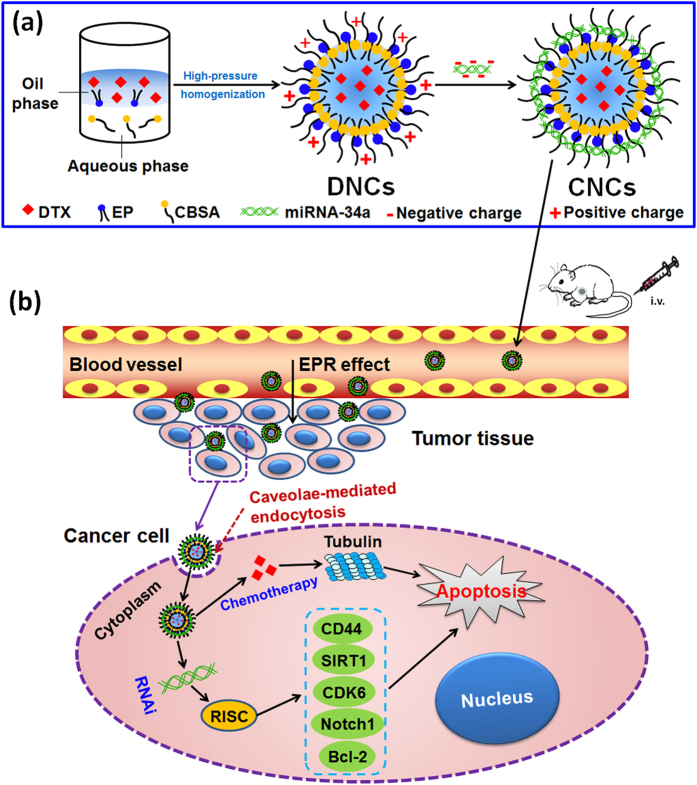
Schematic illustration of core-shell nanocarriers for the co-delivery of DTX and miRNA-34a. (**a**) Preparation of the CNCs. (**b**) Proposed mechanism for the cancer cell killing. The CNCs enter the blood vessel via systemic administration. Then, they accumulate in tumor tissue through the EPR effect, are taken up by cancer cells, obtain cytosolic delivery, and finally induce cell apoptosis via the synergistic anticancer effect of DTX and miRNA-34a.

**Figure 2:**
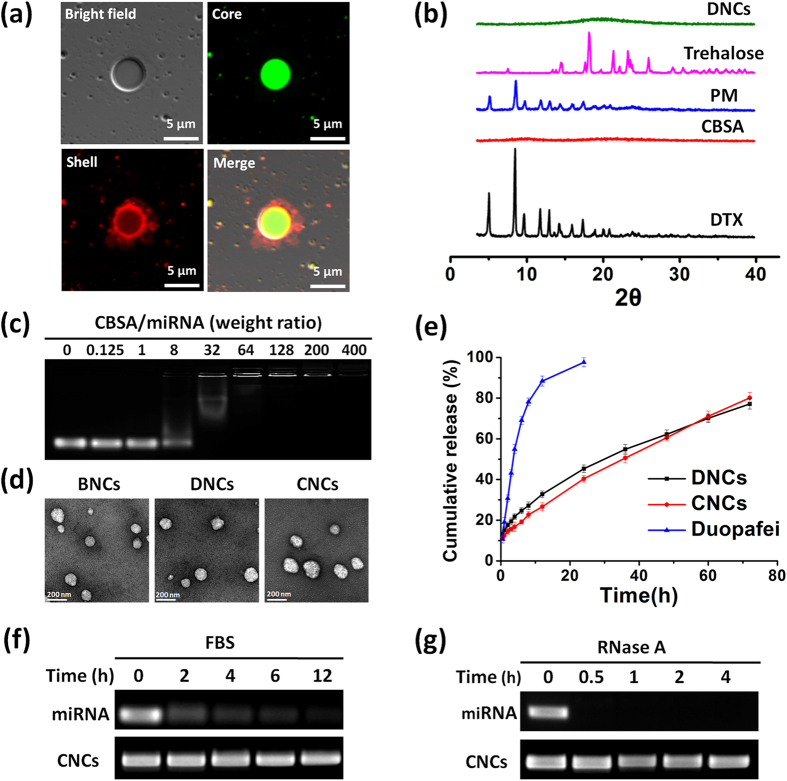
Characterization of core-shell nanocarriers for co-delivery of DTX and miRNA-34a. (**a**) Core-shell structure of nanocarriers confirmed by CLSM. The core was loaded with C6 (green) and the shell was labeled with RBITC (red). Scale bar: 5 μm. (**b**) Physical state of the drug in the core-shell nanocarriers analyzed by PXRD. The DNCs sample was obtained by freeze-drying. Trehalose was the protectant during freezing. PM: the physical mixture of DTX and CBSA. (**c**) RNA binding ability of CBSA in core-shell nanocarriers investigated by agarose gel electrophoresis. The lane numbers were the mass ratios of CBSA/miRNA. (**d**) TEM images of BNCs, DNCs and CNCs. Scale bar: 200 nm. (**e**) *In vitro* DTX release from Duopafei^®^, DNCs and CNCs. (**f**) Serum stability study of miRNA-34a protected by CNCs investigated by agarose gel electrophoresis. (**g**) Protection of miRNA-34a from RNase A degradation evaluated by agarose gel electrophoresis.

To co-deliver DTX and miRNA-34a, DTX was encapsulated in the lipid core of nanocarriers, while miRNA-34a was self-assembled in the shell of the nanocarriers via electrostatic interactions with CBSA (as illustrated in Fig. 1a). The encapsulation efficiency (EE) and drug loading (DL) of DTX determined by HPLC were 83.46 ± 2.36% and 13.91 ± 0.39%, respectively. To confirm that DTX was well encapsulated in the cores of the nanocarriers, powder X-ray diffraction (PXRD) examination was carried out. As shown in Fig. 2b, characteristic diffraction peaks of DTX from pure drug particles (DTX) and physical mixture (PM) are displayed; in contrast, the diffraction peaks of DTX disappear in the diffractogram of freeze-dried DTX-loaded nanocarriers (DNCs). This result therefore suggested that the drug present as the amorphous state and was well incorporated into the cores of DNCs without drug leaky. The good drug encapsulation was further identified by differential scanning calorimetry (DSC) analysis (Supplementary Fig. S3). The RNA binding ability of CBSA in the nanocarriers was investigated by agarose gel electrophoresis. As shown in Fig. 2c, as the weight ratio of CBSA/miRNA increased to 64, the migration of miRNA-34a was completely retarded, therefore indicating that all of the miRNA-34a was completely condensed by the CBSA in the nanocarriers at or above this ratio. Subsequent studies were performed with the nanocarriers prepared at the weight ratio of 64.

The average particle size of DTX and miRNA-34a co-loaded nanocarriers (CNCs) was approximately 183.9 ± 2.8 nm with polydispersity index (PDI) value less than 0.2 (see Supplementary Fig. S4 and Table S1). Transmission electron microscope (TEM) examination displays spherical particles with a diameter of 150–200 nm, which is in line with dynamic light scattering (DLS) results (Fig. 2d). The zeta potential of the blank core-shell nanocarriers (BNCs) was approximately 29 mV, and upon loading miRNA-34a, the surface positive charge decreased to approximately 23 mV for neutralization. These results demonstrated that the nanocarriers could successfully load DTX and miRNA-34a and were stable enough against aggregation.

The *in vitro* release profile of DTX from DNCs and CNCs was investigated using a dialysis method. As shown in Fig. 2e, almost all of the DTX in Duopafei^®^ was released within 24 h; in contrast, approximately 80% of the DTX was released from the DNCs or CNCs within 72 h, thereby exhibiting a sustained release profile. No significant difference in the release behavior was observed between the DNCs and the CNCs, indicating that the absorption of miRNA-34a had little influence on the drug release from the nanocarriers.

### Protection of miRNA-34a from serum and RNase degradation

miRNAs are very unstable in nuclease and serum, which is one of the pressing difficulties in systemic administration for efficient gene silencing. Therefore, it is crucial to prepare carriers that could protect miRNAs from RNase and serum degradation. To assess the role of CNCs in protecting miRNA-34a from degradation in the serum and RNase A, the stability of miRNA-34a was tested by agarose gel electrophoresis. As shown in Fig. 2f, free miRNA-34a was gradually degraded after incubation with FBS, indicating that naked miRNA was unstable in serum. In contrast, the band of miRNA-34a incorporated in CNCs was clearly visible after incubation with fetal bovine serum (FBS) for 12 h. Moreover, CNCs could also enhance the stability of miRNA-34a in RNase A. Figure 2g shows that naked miRNA-34a was rapidly degraded by RNase A after 30 min incubation, while miRNA-34a encapsulated in CNCs remained intact after incubation for 4 h. These results suggested that CNCs can efficiently protect miRNA-34a against serum and nucleases degradation.

### Cellular uptake

**Figure 3:**
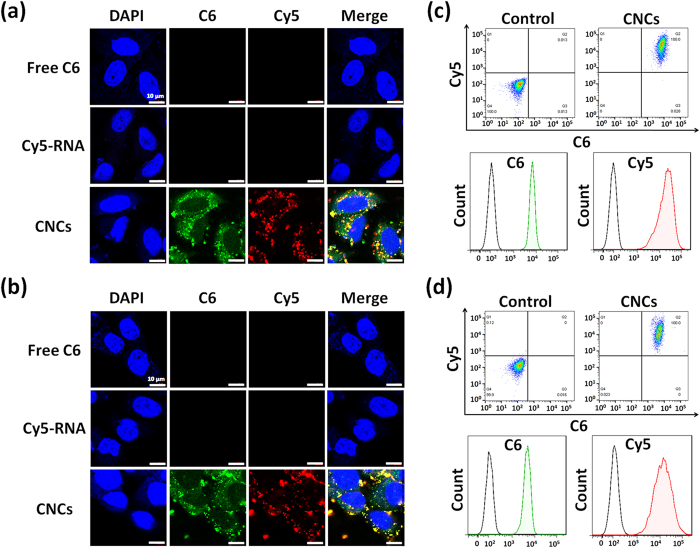
Cellular uptake of CNCs observed by CLSM (**a**,**b**) and measured by FCM (**c**,**d**). Dual fluorescence-labeled CNCs were prepared by loading C6 (green) in the cores and Cy5-RNA (red) in the shells of CNCs. A549 cells (**a**,**c**) or 4T1 cells (**b**,**d**) were incubated with CNCs for 4 h at 37 °C. Cell nuclei (blue) were stained with DAPI. Free dye (C6 or Cy5-RNA) was used as control. Scale bar: 10 μm.

To detect by CLSM and flow cytometry (FCM), fluorescence probes, C6 (green) was loaded in the core and Cy5-labeled miRNA-34a (Cy5-RNA, red) was incorporated in the shell of CNCs simultaneously. To evaluate the stability of C6 and Cy5-RNA in CNCs, the *in vitro* leakage of C6 and Cy5-RNA from CNCs in serum-free medium were performed. As shown in Supplementary Fig. S5, less than 3% of C6 or Cy5-RNA were released from CNCs at 6 h, thereby indicating that the fluorescence-labeled CNCs were stable during use. As shown in Fig. 3a and b, after 4 h incubation with free C6 and Cy5-RNA, little fluorescent signals were present in A549 or 4T1 cells, indicating that free C6 or Cy5-RNA could hardly be internalized by cells. In contrast, significant fluorescent signals of C6 (green in the web version) and Cy5-RNA (red in the web version) were present in the cytoplasm of A549 or 4T1 cells upon treated with dual fluorescence-labeled CNCs; moreover, obvious yellow fluorescent spots in the cytoplasm after overlapping of green and red fluorescence could be seen in the merged images. FCM analysis further demonstrated that almost 100% of 4T1 or A549 cells were both C6-positive and Cy5-positive (Fig. 3c and d). These results suggested that CNCs could be efficiently taken by 4T1 or A549 cells and were able to deliver drugs and miRNAs into the same tumor cells simultaneously.

**Figure 4:**
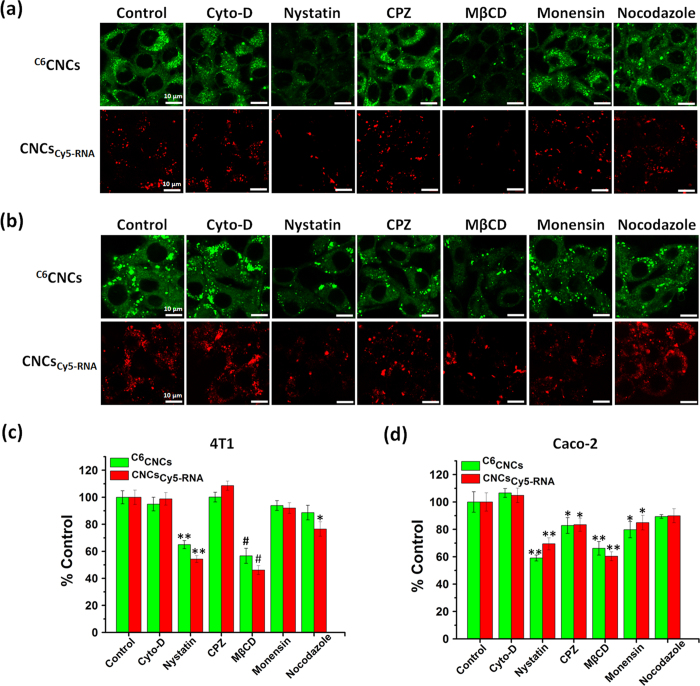
Internalization mechanism of the CNCs in 4T1 and Caco-2 cells. CLSM images of intracellular fluorescence of CNCs in (**a**) 4T1 cells and (**b**) Caco-2 cells pretreated with various inhibitors at 37 °C. Scale bar: 10 μm. Relative cellular uptake percentage calculated from the mean fluorescence intensity measured by FCM in (**c**) 4T1 cells and (**d**) Caco-2 cells. CNCs were labeled by C6 (green) or Cy5-RNA (red). The cells incubated with only CNCs without inhibitors represented the control, and the related data was set as 100%. *P < 0.05, **P < 0.01, ^#^P < 0.001 versus control.

To study the endocytosis pathways of CNCs, various endocytosis inhibitors were utilized in the incubation with 4T1 or Caco-2 cells, and the cellular uptake of CNCs was determined by both CLSM and FCM. The inhibitors include cytochalasin-D (Cyto-D), nystatin, chlorpromazine (CPZ), methyl-β-cyclodextrin (MβCD), monensin and nocodazole, which suppress macropinocytosis, caveolae-mediated pathways, clathrin-mediated routes, lipid-raft mediated pathways, lysosome-involved internalization and microtubule-related internalization, respectively^25^,^26^. As shown in Fig. 4 and Supplementary Fig. S6, among these inhibitors, the inhibition effect of MβCD and nystatin on the internalization of CNCs was most remarkable for 4T1 cells, indicating that the internalization of CNCs in 4T1 cells was related to the lipid raft and caveolae-mediated pathways. The cellular uptake of CNCs in Caco-2 cells mainly related to the caveolae-mediated pathway, lipid-raft mediated pathway, and clathrin-mediated pathway, but the suppression by nystatin and MβCD was the most profound.

### Caveolae-mediated cellular internalization, intracellular trafficking and tumor spheroid penetration

**Figure 5:**
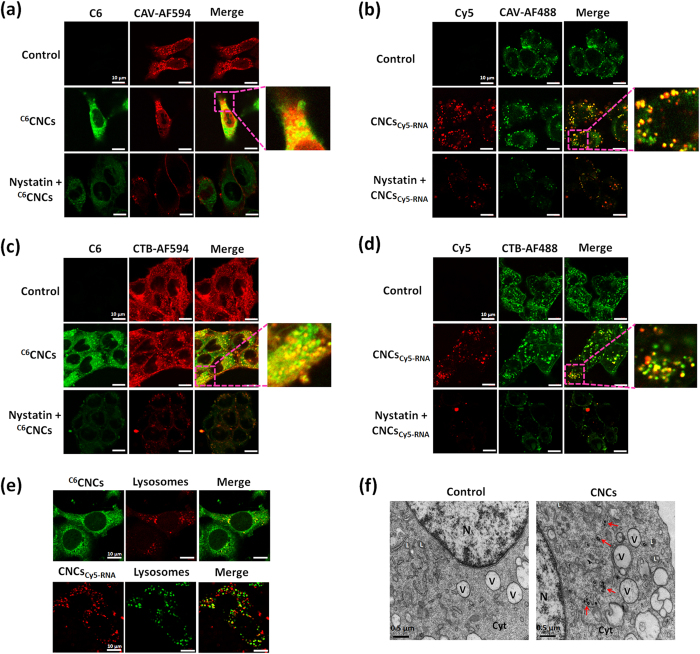
Caveolae-mediated cellular internalization and intracellular trafficking of CNCs in 4T1 cells. Colocalization of (**a**) ^C6^CNCs or (**b**) CNCs_Cy5-RNA_ with caveolae after incubation for 4 h at 37 °C in the absence or presence of nystatin. Caveolin-1 (CAV-1) was marked by anti-caveolin-1 antibody/Alexa Fluors^®^ 594 (red) or 488 (green). Colocalization of (**c**) ^C6^CNCs or (**d**) CNCs_Cy5-RNA_ with CTB-Alexa Fluors^®^ 594 (red) or 488 (green) after incubation for 4 h at 37 °C in the absence or presence of nystatin. Yellow spots indicate the colocalization of CNCs with caveolae or CTB. 4T1 cells without treatment were used as the control. Scale bar: 10 μm. (**e**) Colocalization of CNCs with lysosomes after incubation for 4 h at 37 °C. Scale bar: 10 μm. (**f**) Cytosolic location of the CNCs in 4T1 cells observed by TEM after incubation for 4 h at 37 °C. Control group was the normal cells without treatment. Red arrow heads indicate CNCs. Cyt, cytoplasm; N, nucleus; V, vesicle; L: lysosome. Scale bar: 0.5 μm.

The caveolae-mediated cellular internalization of CNCs in 4T1 and Caco-2 cells was further demonstrated using anti-caveolin-1 antibody-Caveolae Marker (Alexa Fluors^®^ 488 or 594) and the pathway marker of caveolae-mediated internalization, cholera toxin subunit B (CTB)-Alexa Fluor^®^ 488 (or 594). Figure 5a and b shows the colocalization of CNCs with caveolae in 4T1 cells. The yellow areas are from the merged images, indicating the high colocalization of the CNCs with caveolae. In contrast, the cellular internalization of CNCs decreased significantly in the presence of nystatin, an inhibitor that can deplete cholesterol from membranes and block the formation of caveolae. Figure 5c and d shows the high colocalization of CNCs with the pathway marker of caveolae-mediated internalization, CTB. The addition of nystatin significantly inhibited the cellular uptake of the CNCs and CTB. The caveolae-mediated cellular internalization of CNCs was further confirmed in Caco-2 cells, intestinal epithelial cells possessing abundant caveolaes^27^. As shown in Supplementary Fig. S7, similar results were observed in Caco-2 cells. These results suggested that the CNCs could be internalized via the caveolae-mediated pathway.

In general, the caveolae-mediated pathway can enable the nanoparticles to gain cellular entry without entrapment in lysosomes and therefore avoid lysosomal degradation^23^,^28^. Hence, a study of the intracellular trafficking of CNCs in lysosomes was performed. After overlaying the images of lysosomes marked by Lysotracker Red or Green with the CNCs labeled by C6 (green) or Cy5-RNA (red), a few yellow spots in the merged images are observed (Fig. 5e). This result demonstrated that a few CNCs were detained by lysosomes as they entered the cells. To directly observe the distribution of the CNCs in cells, TEM examination of the 4T1 cells after incubation with the CNCs for 4 h was carried out. Significantly, the CNCs distributed in the cytoplasm rather than in the lysosomes or closed vesicles (Fig. 5f).

Since the 2D cell culture models cannot fully represent tumors *in vivo*, to better mimic the real solid tumor condition *in vivo*, 4T1 multicellular tumor spheroids were established to evaluate the penetration of CNCs in tumor spheroids. As shown in Supplementary Fig. S8, free C6 and Cy5-RNA could hardly penetrate into tumor spheroids. In contrast, after incubation with dual fluorescence-labeled CNCs, considerable fluorescent signals of C6 (green) or Cy5-RNA (red) were present in the tumor spheroid; moreover, remarkable yellow spots were observed after overlapping the green and red fluorescence in the merged image. These results demonstrated that the CNCs had excellent tumor penetration ability and were able to co-deliver small molecule drugs and miRNAs into tumor spheroids simultaneously.

### *In vitro* RNAi experiments

**Figure 6:**
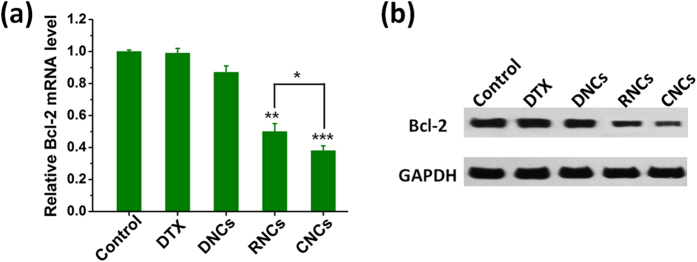
Suppression of anti-apoptosis Bcl-2 expression in 4T1 cells by transfecting with miRNA-34a in CNCs. (**a**) Expression of Bcl-2 mRNA detected by qRT-PCR. The cells without treatment represented the control and the related data was set as 1.0. *P < 0.05, **P < 0.01, ***P < 0.001 versus control. (**b**) Expression of Bcl-2 protein detected by western blot assay. GAPDH was used as an internal control to normalize protein expression. The cells without treatment represented the control.

The miRNA-34a transfection efficiency was analyzed based on the gene silencing efficiency at both the transcription and protein levels. Bcl-2 can prevent cell death via the mitochondrial apoptosis pathway, and overexpression of Bcl-2 would render the tumor cells resistant to anticancer drugs. Therefore, down-regulation of Bcl-2 could inhibit tumor growth and increase sensitivity to chemotherapy^29^,^30^. Bcl-2 is considered as one of the promising down-regulation targets of miRNA-34a^31^. In the present study, the expression of Bcl-2 was detected after the restoration of miRNA-34a by CNCs at both the transcription and translation levels by qRT-PCR and western blot, respectively. As shown in Fig. 6, compared with the control cells, the expression of Bcl-2 in the 4T1 cells treated with DTX and DNCs exhibited no significant difference; in contrast, remarkable down-regulation of Bcl-2 expression in cells was observed upon treatment with miRNA-34a loaded nanocarriers (RNCs) or CNCs, along with better inhibition effect from CNCs. These results suggested that the core-shell nanocarriers mediating the intracellular delivery of miRNA-34a could efficiently suppress the expression of Bcl-2.

### *In vitro* cytotoxicity and apoptosis

**Figure 7:**
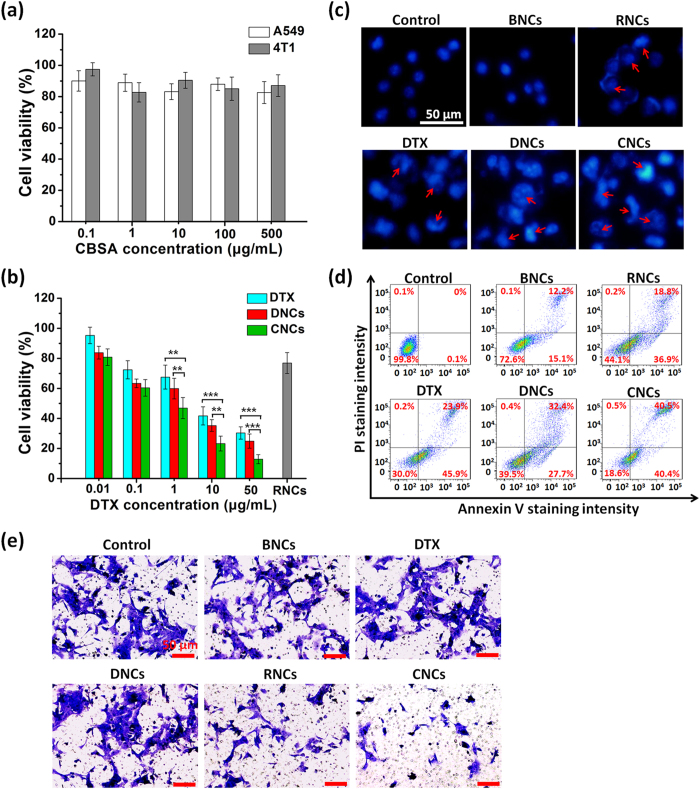
*In vitro* antitumor efficacy. (**a**) Cell cytotoxicity of 4T1 cells and A549 cells treated with BNCs of different concentrations at 37 °C. (**b**) Cell cytotoxicity of 4T1 cells treated with different formulations at 37 °C. The concentration of miRNA-34a was fixed at 100 nM. *P < 0.05, **P < 0.01, ***P < 0.001. (**c**) Cell apoptosis induction and morphological changes in 4T1 cells treated with different formulations imaged by fluorescence microscopy. Cell nuclei were stained with DAPI. The concentrations of miRNA-34a and DTX were 100 nM and 10 μg/mL, respectively. The control group was the normal cells without treatment. Red arrow heads indicate the typical apoptosis features such as chromatin condensation, fragmentation and apoptotic body formation. Scale bar: 50 μm. (**d**) Cell apoptosis detected by FCM using an AnnexinV-FITC/PI apoptosis detection kit. (**e**) Inhibition migration of 4T1 cells examined by transwell chamber assay at 37 °C. Blue regions indicate the migrated cells. The control group was the normal cells without treatment. Scale bar: 50 μm.

The *in vitro* cytotoxicity of CNCs was evaluated by MTT assay. As shown in Fig. 7a, the viability of A549 and 4T1 cells treated with BNCs was higher than 80% as the concentration of CBSA changes from 0.1 to 500 μg/mL, indicating that the blank nanocarriers had low cytotoxicity under the tested concentrations. Free DTX, DNCs and CNCs exhibited concentration dependent cytotoxicity on 4T1 cells (Fig. 7b). DNCs showed slightly higher cytotoxicity than free DTX at the same drug concentration, which could be attributed to the higher uptake efficiency of drugs by nanocarriers. The cells treated with RNCs displayed a reduced viability at a fixed miRNA-34a concentration of 100 nM. Although miRNA-34a can inhibit cell proliferation, it cannot induce strong cytotoxicity completely. However, compared with free DTX, DNCs or RNCs at the same dose, significant decreases of cell viability were observed in the cells treated with the CNCs, suggesting the synergistic cytotoxicity by co-delivery of DTX and miRNA-34a.

To evaluate the cell apoptosis induced by the CNCs in 4T1 cells, the nuclei stained with DAPI were observed by fluorescence microscopy. Cell apoptosis was evaluated according to the nuclear morphology changes, including chromatin condensation, fragmentation and apoptotic body formation. As depicted in Fig. 7c, the nuclei from the control group without treatment were homogeneously stained, appearing spherical and intact with bright blue fluorescence. The cells treated with free DTX, the DNCs and the RNCs displayed apoptotic features such as chromatin condensation and fragmentation. Importantly, the cells treated with the CNCs had the strongest typical features of apoptosis, indicating the greatest cell apoptosis. To evaluate the cell apoptosis quantitatively, AnnexinV-FITC/PI apoptosis detection kits were employed. As shown in Fig. 7d, free DTX, DNCs and RNCs were able to induce cell apoptosis more efficiently compared with the control group. Moreover, the highest cell apoptosis was observed from the cells treated with CNCs co-loaded DTX and miRNA-34a.

### Inhibition of tumor cell migration

The treatment failure for breast cancer mainly stemmed from metastasis-related recurrence; therefore, the inhibition of tumor cell migration is an effective strategy for cancer treatment by preventing tumor metastasis. In the present study, the inhibition of 4T1 cell migration by the CNCs was evaluated by transwell chamber assay. As shown in Fig. 7e and Supplementary Fig. S9, the cells without treatment or treated with BNCs exhibited high migration ability. DTX and DNCs had slight inhibition effects, whereas the migration of cells treated with RNCs and CNCs was significantly suppressed, suggesting that miRNA-34a could induce high inhibition of tumor cell migration, which was consistent with previous reports^32^. Notably, CNCs had a more profound inhibition effect than RNCs. These results indicated the synergistic effect on the inhibition of tumor cell migration via co-delivery of DTX and miRNA-34a.

### Pharmacokinetics, *in vivo* imaging and biodistribution

The plasma concentration-time profiles of DTX in rats after intravenous administration of Duopafei^®^ (free drug) and DNCs are shown in Supplementary Fig. S10, and the main pharmacokinetic parameters are depicted in Supplementary Table S2. The results demonstrated the significant differences in the pharmacokinetic profiles for Duopafei^®^ and DNCs. The DTX plasma concentration was still detectable at 12 h after injection of DNCs, while it was no longer measurable at 8 h upon injection of Duopafei^®^. Compared with Duopafei^®^, the area under the plasma concentration–time profiles (AUC), t_1/2_ and the mean residence time (MRT) from the DNCs increased by approximately 5.8, 3.0 and 2.7 times, respectively, and the total plasma clearance (CL) decreased remarkably, indicating enhanced bioavailability and prolonged circulation time.

**Figure 8:**
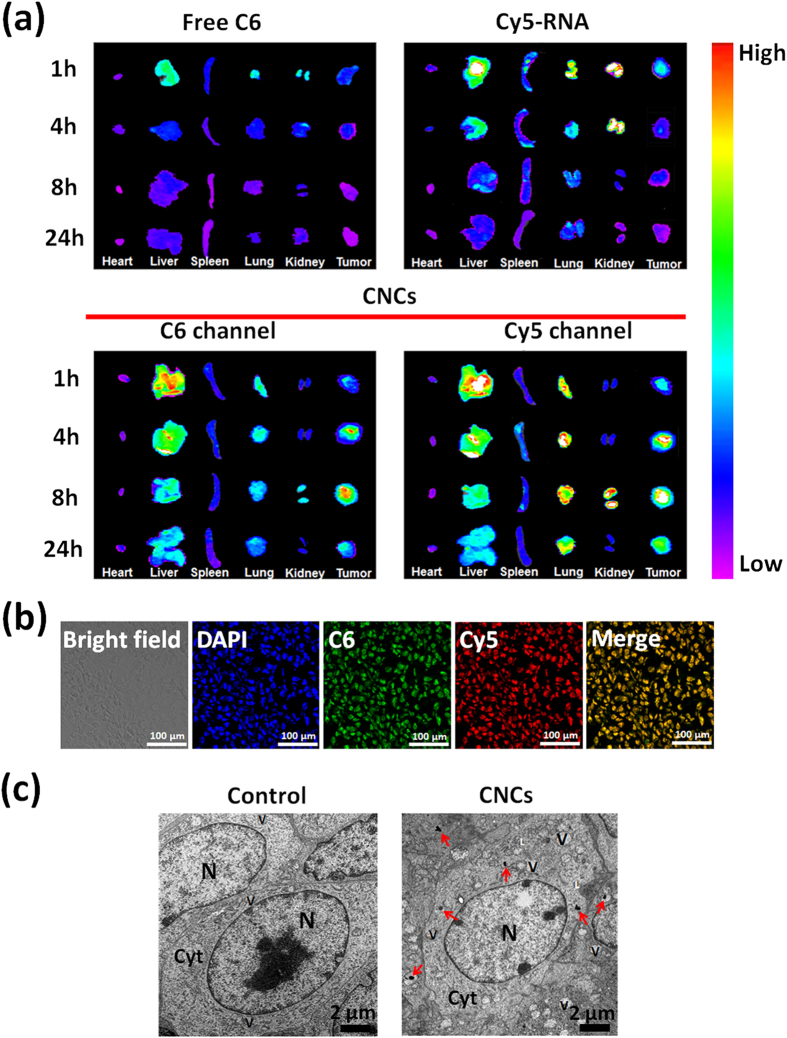
*In vivo* imaging and biodistribution of CNCs in 4T1 tumor-bearing mice. (**a**) *Ex vivo* fluorescence images of heart, lung, kidney, spleen, tumor and liver harvested from the mice at 1 h, 4 h, 8 h and 24 h after injection. (**b**) Biodistribution of dual fluorescence-labeled CNCs in tumors harvested from tumor-bearing mice at 4 h post-injection. Cell nuclei were stained with DAPI. C6: green; Cy5-RNA: red; Scale bar: 100 μm. (**c**) TEM images of sections from isolated tumor collected at 4 h post-injection. The control group was the tumor harvested from tumor-bearing mice without treatment. Red arrow heads indicate the distribution of the CNCs. Cyt, cytoplasm; N, nucleus; V, vesicle; L: lysosome. Scale bar: 2 μm.

The biodistribution of CNCs was investigated in 4T1 tumor-bearing mice using an *in vivo* imaging system. As shown in Fig. 8a and Supplementary Fig. S11, most of the free C6 and Cy5-RNA were distributed in the liver after injection and rapidly eliminated from the body. In contrast, significant fluorescent signals of C6 and Cy5 were detected at 1 h post-injection of the CNCs. Moreover, the fluorescence intensity at the tumor sites increased gradually with elapsed time, indicating the accumulation of CNCs in tumor sites. *Ex vivo* fluorescence images of isolated organs provided results that are consistent with those of *in vivo* imaging. The fluorescent intensity from free C6 and Cy5-RNA groups declined rapidly, and the tumor sites showed little accumulation after injection. In contrast, upon injection of CNCs, the fluorescent intensity of the tumor increased gradually and reached a peak at 8 h post-injection. The effective accumulation of CNCs in the tumors could be ascribed to the prolonged circulation time and the EPR effect.

To demonstrate the co-delivery of drugs and miRNA-34a by CNCs to tumors, CLSM and TEM examinations were employed to observe the sectioned tumor tissues. Green (C6) or red (Cy5-RNA) fluorescence are displayed in the tumor sections, and yellow fluorescence was observed in the merged pictures (Fig. 8b), indicating that the CNCs could penetrate into the tumors with co-delivery of both drugs and miRNA-34a. Meanwhile, the distribution of the CNCs in tumors was further demonstrated by the TEM examinations. As shown in Fig. 8c, the nanoparticles were clearly observed in the cytoplasm of the tumor tissues.

### *In vivo* antitumor efficacy

**Figure 9:**
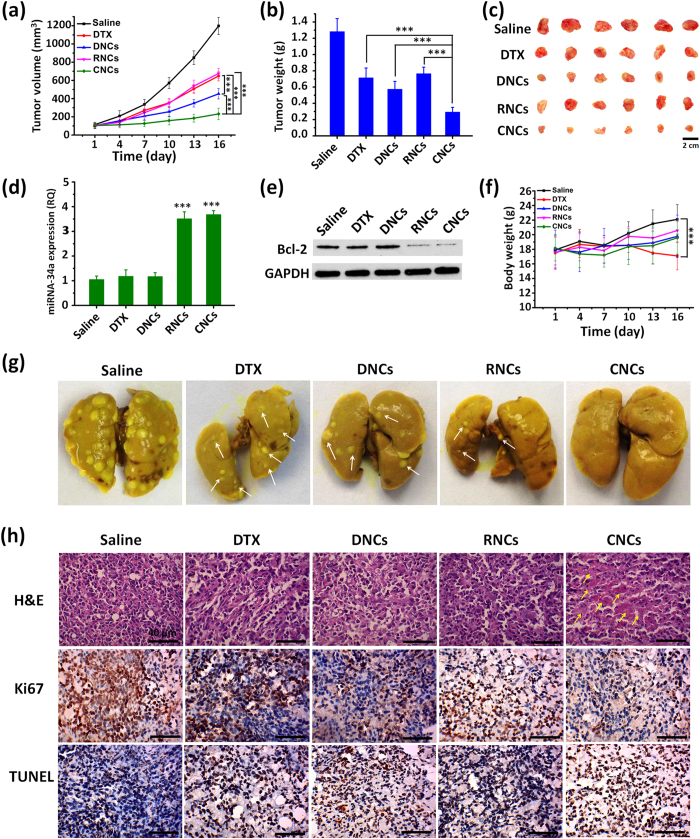
*In vivo* antitumor efficacy in 4T1-tumor bearing mice. (**a**) Tumor growth curves. (**b**) Tumor weight. (**c**) Images of representative tumors excised from mice. (**d**) Intratumoral delivery of miRNA-34a detected by PCR. (**e**) Bcl-2 expression in the tumors investigated by western blot. (**f**) Body weight changes. (**g**) Tumor burdens in the excised lungs. White arrow heads indicate tumor burdens. (**h**) H&E, Ki67 and TUNEL immunohistochemical analysis of harvested tumors. Yellow arrow heads indicate examples of positive tumor suppression with H&E staining. Brown staining indicates positive cells for Ki67 and TUNEL. Scale bar: 40 μm. *P < 0.05, **P < 0.01, ***P < 0.001.

The *in vivo* antitumor efficacy of different formulations was evaluated on 4T1 tumor-bearing mice models, in terms of tumor volumes, body weight changes, tumor weight, numbers of tumor burdens in the excised lungs and immunohistochemical analysis of the isolated tumors. Among these formulations, the CNCs suppressed the tumor growth most efficiently with regard to the tumor volume and weight (Fig. 9a,b and c). The inhibitory rates of DTX, DNCs, RNCs and CNCs calculated from the tumor weight were 44.3%, 55.2%, 40.3% and 77.1%, respectively, indicating the superior anticancer effect of the CNCs. Compared with DTX, the DNCs showed a better inhibition effect on tumor growth (P < 0.001), which might be ascribed to the efficient delivery of drugs to tumor cells by core-shell nanocarriers. Moreover, the PCR and western-blot results showed that RNCs and CNCs were able to efficiently deliver miRNA-34a to tumors (Fig. 9d) and suppress the anti-apoptotic Bcl-2 expression at the protein level (Fig. 9e).

The 4T1 tumor metastasis model has been widely used to evaluate metastatic cancer by visible metastatic nodules formed in lungs^33^. Hence, the tumor burdens in the lungs obtained from the 4T1 tumor-bearing mice were analyzed after the therapy schedule was finished. As depicted in Fig. 9g and Fig. S12a, the tumor burdens with an average number of 41.3 were present in the lungs of the saline group, indicating the high metastasis of 4T1 tumor cells; in contrast, significant reductions in the number of tumor nodules were found in the other groups. Importantly, the number of tumor burdens from the groups treated with the miRNA-34a-loaded formulations, RNCs and CNCs, was less than those of the other formulations, and few tumor burdens were visualized from the group treated with the CNCs. These results indicated that miRNA-34a played a vital role in the inhibition of tumor metastasis, and the co-delivery of DTX and miRNA-34a made this inhibition more profound.

To further confirm the anticancer efficacy, the tumors were excised, and immunohistochemical analysis including H&E, TUNEL and Ki67 were performed (Fig. 9h, Supplementary Fig. S12b and c). H&E staining showed that the tumor from the saline group was hypercellular with no obvious necrosis or apoptosis, and the nuclei were stained dark blue. In contrast, the largest remission of tumor cells with significant necrosis and apoptosis was observed in the group treated with the CNCs. Moreover, as shown in the results of the TUNEL analysis, among these formulations, the CNCs exhibited the highest percentage of apoptotic cells. Ki67 analysis of cell proliferation further confirmed the consistent results. Therefore, these results suggested that CNCs induced superior cancer cell apoptosis and efficiently blocked proliferation *in vivo*.

No serious body weight loss was observed in the mice treated with the DNCs, RNCs and CNCs during the experiments, indicating that the nanocarriers caused no severe systemic toxicity *in vivo* (Fig. 9f). However, the body weight of mice treated with DTX showed a decreasing trend, which might be associated with the toxicity of the nonionic-surfactant Tween-80 in the DTX formulations (Duopafei^®^). The *in vivo* safety of the nanocarriers was further investigated by H&E staining and CD68 immunohistochemical analysis on normal mice. As shown in Fig. S13, compared with saline and DTX, the nanocarrier groups did not induce obvious inflammation and pathological changes, such as lymphocytic infiltration, microgranulation, cell degeneration and necrosis. These results suggested that the core-shell nanocarriers were of low toxicity and would be safe for administration *in vivo*.

## Discussion

We have demonstrated that the core-shell nanocarriers with good biocompatibility could successfully co-deliver chemotherapeutic agents and gene or protein drugs. Generally, most chemotherapeutic drugs are poorly water-soluble, while miRNAs are polyanionic molecules with high water-solubility, instability and high molecular weight. In the present study, DTX and miRNA-34a, two anticancer agents with completely different physicochemical properties, were successfully encapsulated in the cores and shells of the nanocarriers, respectively. The cores of the nanocarriers composed of oleoyl macrogolglycerides, Labrafil^®^ M 1944 CS, which is a good solvent for many poorly water-soluble drugs, such as paclitaxel^34^, morin^35^, cyclosporine A^36^, etc. Meanwhile, the cationic albumin in the shells of the nanocarriers provides positive charges for the encapsulation of therapeutic genes or negatively charged protein drugs by electrostatic interaction. Furthermore, the rich amino and carboxyl groups of cationic albumin also provide the possibility for further functional modification, such as PEGylation, targeting ligand linkage and drug conjugation. Albumin, approved by FDA as a versatile material for drug delivery systems, possesses three domains composed of hydrophobic amino acids, thereby offering extraordinary ligand binding capacity for hydrophobic compounds^37^,^38^. To condense negatively charged miRNAs, the side-chain of albumin was modified with ethylenediamine via amide bond to obtain positive charges without destroying its sequence structure and properties^39^. Indeed, the encapsulated miRNA-34a in the core-shell nanocarriers displayed excellent protection against degradation by RNase and serum. On the other side, cationic polymers employed as materials for drug delivery system, such as PEI, would generate potential toxicity to the human body^40^. The nanocarriers coated with CBSA induced no obvious inflammation or pathological changes in the BALB/c mice models as demonstrated by H&E and CD68 immunohistochemical analysis, which is in line with a previous report that CBSA possessed excellent biocompatibility^39^. Additionally, compared with conventional co-delivery systems, the preparation procedure for nanocarriers was simple without complicated synthesis processes, implying their potential for industrial production and clinical application.

The present core-shell nanocarriers entered cells via the caveolae-mediated pathway without entrapment within lysosomes and thus efficiently improved the utilization of their cargoes by avoiding lysosomal degradation. A number of drugs exert their activities in the cells, predominantly in the cytosol. Therefore, nanoparticles that obtain cell entry via caveolae-mediated endocytosis and bypass endosomes/lysosomes are attracting increasing attention due to their enhanced intracellular delivery of protein and gene drugs^41^,^42^,^43^. In the present study, after blocking the caveolae-mediated pathway by nystatin, the uptake of the nanocarriers by the 4T1 and Caco-2 cells were remarkably reduced. The high colocalization of nanocarriers with caveolae and CTB further verified the participation of caveolae in the internalization process of the nanocarriers. Little colocalization of CNCs with lysosomes and the cytoplasm distribution of the CNCs was observed by CLSM and TEM. It has been reported that albumin-coated nanoparticles could transport myeloperoxidase into cells via caveolae-related pathways because albumin could specifically bind to the protein localized in caveolae and thus alter the internalization pathway^44^,^45^. Our group also found that nanoparticles coated with β-lactoglobulin, a globular protein similar to albumin, penetrated cell membranes into the cytosol via a caveolae-dependent pathway through the interaction between the specific hydrophobic sites of the protein and the cell membranes^24^,^25^. It was thus speculated that albumin-coating on the surfaces of CNCs predominantly contributed to the participation of caveolae in the cellular uptake of CNCs.

The *in vivo* pharmacokinetics and biodistribution performances of the drugs loaded in the core-shell nanocarriers were improved significantly, as indicated by the prolonged MRT, increased AUC and enhanced tumor accumulation. Rapid clearance from the body is one of the inherent drawbacks of free drugs; as a result, frequent administration and higher drug doses are required, thus leading to poor compliance and serious side effects. In contrast with the free drugs, the pharmacokinetic parameters of AUC, t_1/2_ and MRT of DTX encapsulated in the DNCs were increased by approximately 5.8-, 3.0- and 2.7-fold, respectively, and the CL from the blood decreased significantly. The improved pharmacokinetic profile of DTX by the present nanocarriers may be ascribed to the albumin coating. Albumin could protect nanoparticles from being opsonized and consequently reduce their sequestration by the macrophage phagocytic system, thereby obtaining decreased clearance rates and prolonged blood circulation^46^. Moreover, albumin possesses high affinity to a number of cell surface proteins that are expressed in solid tumors, including FcRn, gp18, gp60 and SPARC^37^,^46^. Accordingly, the enhanced tumor accumulation of CNCs mainly resulted from the prolonged circulation time, EPR effect and specific binding of albumin with cancer cells. Meanwhile, the cationic modification of albumin, which endowed positive charges on the surfaces of the CNCs, also promoted their internalization in tumor cells^47^.

Synergistic therapy for metastatic breast cancer was obtained via co-delivery of miRNA-34a and DTX with CNCs. The significant improvement of the anticancer efficacy was ascribed to the complementary mechanisms of the two drugs and efficient co-delivery using the present nanocarriers. DTX is a first-line anticancer drug for clinical treatment of various cancers; however, its treatment effect was greatly compromised by the occurrence of serious adverse effects and the development of drug resistance. miRNA-34a induces apoptosis, blocks proliferation and inhibits cancer stem cell growth and differentiation by down-regulating the tumor-related genes such as Bcl-2^12^,^13^,^48^. Notably, the overexpression of Bcl-2 also enables the tumor cells to resist against anticancer drugs^49^,^50^. Metastasis-related recurrence is the main cause of death for breast cancer patients in clinic. Moreover, miRNA-34a inhibits the EMT of the cancer cells by suppressing the expression of the EMT-inducing transcription factor and impacting the IL-6 R/STAT3/miR-34a feedback loop, which helps inhibit the migration of breast cancer cells and metastasis^51^,^52^. Therefore, the combination of DTX and miRNA-34a aiming to produce synergistic treatment effects for breast cancer is highly desired. However, both DTX and miRNA-34a act in cytosol and thus simultaneously delivering the two drugs to cytosol is vital. miRNA-34a tends to be degraded by lysosomes as it internalizes in cells. In this study, the CNCs could simultaneously load the two drugs and deliver them into the cytosol via caveolae endocytosis without entrapment in lysosomes. The expression of Bcl-2 at both the transcription and protein levels *in vitro* and *in vivo* was efficiently inhibited by the CNCs; this inhibition effect increased the sensitivity of the cancer cells to DTX and subsequently induced enhanced apoptosis. Moreover, cancer cell migration and metastasis were suppressed significantly, as indicated by the results of *in vitro* transwell migration experiments and lung metastasis in 4T1 tumor-bearing mice. Therefore, this study demonstrated that the combination of DTX and miRNA-34a resulted in enhanced treatment for metastatic breast cancer.

In summary, we have developed a strategy for the co-delivery of DTX and miRNA-34a using a core-shell nanocarrier for the treatment of metastatic breast cancer. Importantly, the nanocarrier could co-deliver the two drugs into tumor cells via a caveolae-mediated pathway without entrapment in endosomes/lysosomes, thus obtaining efficient utilization of the drugs. Compared to DTX or miRNA-34a formulations used alone, the co-delivery of the two drugs with the nanocarrier obtained improved anticancer efficacy by inhibiting tumor growth and metastasis through their complementary mechanisms. Overall, this strategy provides a new nanoplatform for the combination of insoluble drugs and gene or protein drugs, and holds a great potential for the treatment of metastatic breast cancer.

## Methods

Please refer to the Supplemental Information for the other Methods.

### Preparation of core-shell nanocarriers

BNCs were prepared via high-pressure homogenizing as reported previously by our group^34^. First, CBSA should undergo heat-induced denaturation to expose the hydrophobic residues prior to use. Briefly, 30 mL of 1.0% (w/v) CBSA solution was heated in a water bath at 90 °C for 10 min and then cooled to room temperature for further use. Next, 100 mg of egg phospholipid was dissolved in 1 mL of Labrafil^®^ M 1944 CS by ultrasonication. Then, the oil mixture was added to the denatured CBSA solution, dispersed at 10 000 rpm for 60 s with a high-speed disperser (XHF-D, Ningbo Scientz Biotechnology Co. Ltd., China), and immediately followed by homogenization at 800 bar using a high pressure homogenizer (AH-2010, ATS Engineering Inc., Canada) until an equilibrium size was reached.

DNCs were prepared by a similar procedure to the one that was utilized to prepare the BNCs, except DTX and EP were dissolved in 1 mL of Labrafil^®^ M 1944 in advance. The EE and DL of the DNCs was obtained by determining the amount of drug encapsulated in the DNCs. Briefly, the DNCs were incubated with 10% sodium dodecyl sulfate (SDS) to destruct the DNCs, then diluted in methanol and further purified by centrifugation (10 000 rpm, 15 min). The DTX concentration of the supernatant was determined by high-performance liquid chromatography (HPLC) described as below. The EE and DL were calculated using the following formulas: EE (%) = weight of DTX in DNCs/weight of the feeding DTX × 100%; DL (%) = weight of DTX in DNCs/weight of CBSA × 100%.

CNCs were prepared based on electrostatic interactions between CBSA and miRNA-34a. Briefly, 100 μL of a miRNA-34a solution was added rapidly to an equal volume of an aqueous dispersion of DNCs under mixing by vortex and incubated for 30 min at room temperature prior to use. RNCs were prepared by a similar procedure without the addition of DTX as described above. The preparation of dual fluorescence-labeled CNCs loaded with C6 in the core and Cy5-RNA in the shell was the same as the procedure for the CNCs, except that DTX and miRNA-34a were replaced by the fluorescent probes C6 and Cy5-RNA, respectively.

### Cellular uptake and internalization mechanism

The cellular uptake and distribution of CNCs were investigated by CLSM, FCM and TEM. Dual fluorescence-labeled CNCs loaded with C6 in the core and Cy5-RNA in the shell were prepared as described above for the analysis of CLSM and FCM. Briefly, 4T1 or A549 cells were seeded in a confocal special dish (35 mm) at a density of 1 × 10^5^ cells/well and incubated for 24 h at 37 °C to achieve a confluence of approximately 70%. Then, the cells were treated with serum-free medium containing dual fluorescence-labeled CNCs, free C6 or Cy5-RNA. After incubation for 4 h at 37 °C, the media were removed, and the treated cells were washed three times with fresh PBS and fixed with 4% formaldehyde for 10 min at room temperature. Cell nuclei were stained with DAPI for 10 min at room temperature. After washing with fresh PBS three times, the cells were finally observed by CLSM (Zeiss LSM 700, Germany).

For quantitative analysis of the cellular uptake of CNCs by FCM, 4T1 or A549 cells were seeded in 12-well plates at a density of 2 × 10^5^ cells/well and then treated with dual fluorescence-labeled CNCs as described above. Then, the cells were washed three times with PBS, harvested with 0.25% trypsin and then transferred to centrifuge tubes. After centrifugal washing with fresh PBS at least three times, the cells were resuspended in 500 μL PBS and immediately determined by FCM (MACSQuant Analyzer 10, Miltenyi Biotec., Germany).

To visually observe the intracellular distribution of CNCs, TEM analysis was performed as follows^53^. Briefly, 4T1 cells were harvested after treatment as mentioned above and then fixed with 2% glutaraldehyde overnight at 4 °C. Subsequently, the cells were post-fixed with 1% osmium tetroxide for 2 h at 4 °C and dehydrated through immersing in serially diluted aqueous ethanol solutions (50%, 70%, 90% and 100%). Then, the specimens were embedded in epoxy resin and cut into 70–80 nm thick sections by a microtome (Leica CM1950, Germany) at room temperature. After staining with uranyl acetate and lead citrate, the sections were finally examined by TEM.

To verify the internalization mechanism of the CNCs, 4T1 and Caco-2 cells were seeded in 12-well plates at a density of 2 × 10^5^ cells/well. After incubation for 24 h, the cells were pretreated with serum-free medium containing various endocytosis pathway inhibitors for 30 min at 37 °C. The kinds of inhibitors and their concentrations were as follows: Cyto-D (10 μg/mL), nystatin (20 μM), CPZ (10 μg/mL), M-β-CD (5 mM), monensin (200 nM) and nocodazole (20 μM)^25^,^54^. The cells without inhibitor treatment served as the control. Subsequently, the cells were incubated with C6-labeled CNCs (^C6^CNCs) or Cy5-RNA-loaded CNCs (CNCs_Cy5-RNA_) for 4 h at 37 °C. Finally, the cells were collected as described above and finally determined by FCM. For the observation by CLSM, 4T1 or Caco-2 cells were seeded in a confocal special dish (35 mm) at a density of 1 × 10^5^ cells/well and treated as described above.

### *In vivo* antitumor efficacy

The animals used in the experiments received care in compliance with the Principles of Laboratory Animal Care and the Guide for the Care and Use of Laboratory Animals. All the animal experiments were performed in accordance with the protocol approved by the China Pharmaceutical University Institutional Animal Care and Use Committee.

4T1 tumor-bearing mice were randomly divided into five groups with nine mice in each group. The mice were injected with normal saline, Duopafei^®^, DNCs, RNCs and CNCs (DTX of 6 mg/kg and miRNA-34a of 2 mg/kg), respectively, 5 times via the tail vein every 3 days. The body weight and tumor volume were recorded at predetermined time points. The tumor volumes were calculated by the following formula: V = (L × W^2^)/2, where L is the longest diameter and W is the shortest diameter of the tumor. The mice were then sacrificed, and the tumor issues were excised and weighed after the treatment schedule was finished. The lungs extracted from mice were fixed in Bouin’s fixative for 24 h, and the tumor burdens on the lungs were recorded. H&E staining were performed for histological evaluation of the tumors. TUNEL assay was performed to assess the cell apoptosis rate of the tumor cells, and Ki67 staining was carried out to evaluate the cell proliferation rate of the tumor cells.

### Statistics

All of the experiments were performed at least in triplicate. The data were presented as the mean ± SD. The statistical significance was analyzed by one-way analysis of variance (ANOVA). The differences were considered to be statistically significant when the P values were less than 0.05 (P < 0.05).

## Additional Information

**How to cite this article**: Zhang, L. *et al*. Cytosolic co-delivery of miRNA-34a and docetaxel with core-shell nanocarriers via caveolae-mediated pathway for the treatment of metastatic breast cancer. *Sci. Rep.* **7**, 46186; doi: 10.1038/srep46186 (2017).

**Publisher's note:** Springer Nature remains neutral with regard to jurisdictional claims in published maps and institutional affiliations.

## Supplementary Material

Supplementary Information
