# Supplementary material for: Cytosolic co-delivery of miRNA-34a and docetaxel with core-shell nanocarriers via caveolae-mediated pathway for the treatment of metastatic breast cancer
Source: Sci Rep. 2017 Apr 6;7:46186. doi: 10.1038/srep46186 (PMC5382875; doi:10.1038/srep46186)
Supplement: Supplementary Information [file srep46186-s1.pdf]

Supplementary Information

Cytosolic co-delivery of miRNA-34a and docetaxel with core-shell nanocarriers via caveolae-mediated pathway for the treatment of metastatic breast cancer

Li Zhang, Xin Yang, Yaqi Lv, Xiaofei Xin, Chao Qin, Xiaopeng Han, Lei Yang, Wei He*, Lifang Yin*

Department of Pharmaceutics, School of Pharmacy, China Pharmaceutical University, Nanjing 210009, PR China

*Corresponding authors: Wei He and Lifang Yin

E-mail address: weihe@cpu.edu.cn (W. He), lifangyin_@163.com (L. Yin)

Mehods

Materials. DTX was purchased from Jiangsu Yew Pharmaceutical Co., Ltd. (Wuxi, China). Mmu-miRNA-34a mimics and Cy5-labeled miRNA-34a (Cy5-RNA) were purchased from RiboBio Co., Ltd. (Guangzhou, China). Labrafil® M 1944 CS (oleoyl macrogolglycerides) was obtained from Gattefossé (Saint-Priest Cedex, France). Egg phospholipid (EP) was purchased from Sinopharm Chemical Reagent Co., Ltd. (Shanghai, China). 1-Ethyl-3-(3-dimethylaminopropyl)-carbodiimide hydrochloride (EDC), bovine serum albumin (BSA), coumarin-6 (C6), 4,6-diamidino-2-phenylindole (DAPI) and 3-(4,5-dimethyl-2-thiazolyl)-2,5-diphenyl-tetrazolium bromide (MTT) were purchased from Sigma-Aldrich (St. Louis, MO, USA). AnnexinV-FITC/PI apoptosis detection kits were purchased from Beyotime Institute of Biotechnology (Haimen, China). LysoTracker and Cholera Toxin Subunit B (CTB)-Alexa Fluors® 488 (or 594) were obtained from Invitrogen (Eugene, Oregon, USA). Anti-caveolin-1 antibody-Caveolae Marker (Alexa Fluors® 488 or 594) was purchased from Abcam (Cambridge, UK). Fetal bovine serum (FBS), trypsin, Roswell Park Memorial Institute (RPMI) 1640 medium, Dulbecco's modified Eagle's minimal essential medium (DMEM), non-essential amino acids, L-glutamine, penicillin and streptomycin were purchased from Thermo Fisher Scientific Inc. (Waltham, MA, USA). All other chemicals were of analytical grade, commercially available and used without further purification.

Cell culture and animals. Murine breast cancer (4T1), human lung adenocarcinoma (A549) and human colon carcinoma (Caco-2) cells were purchased from KeyGEN Biotech Co., Ltd. (Nanjing, China). 4T1 and A549 cells were cultured in RPMI 1640 medium supplemented with 10% FBS, 100 μg/mL streptomycin and 100 U/mL penicillin. Caco-2 cells were cultured in DMEM supplemented with 10% FBS, 1% non-essential amino acids, 1% L-glutamine, 100 μg/mL streptomycin and 100 U/mL penicillin. All cells were cultured in an incubator at 37 ℃ with a humidified atmosphere of 5% CO_2. Female Sprague–Dawley (SD) rats (200-250 g) and BALB/c mice (18-22 g) were purchased from the College of Veterinary Medicine of Yangzhou University (Yangzhou, China). The animals were fed at the condition of 25 ℃ and 55% of humidity in an SPF

class animal facility. The animals used in the experiments received care in compliance with the Principles of Laboratory Animal Care and the Guide for the Care and Use of Laboratory Animals. All the animal experiments were performed in accordance with the protocol approved by the China Pharmaceutical University Institutional Animal Care and Use Committee.

Synthesis of CBSA. CBSA was synthesized from BSA by modification with ethylenediamine as described previously (**Supplementary Fig. S1**)[1]. Briefly, 5 mL of 20% (w/v) BSA solution was slowly added to 125 mL of an ethylenediamine solution (0.9 M, pH 4.75) under stirring. Subsequently, 200 mg of EDC was added, and then, the mixture was stirred at room temperature for 2 h. The reaction was quenched by the addition of 650 μL acetate buffer (4 M, pH 4.75). Then, the reaction solution was concentrated to approximately 10 mL by a protein concentrator (Thermo Fisher, USA)), followed by dialysis against distilled water using a dialysis bag (MWCO 3500) for 72 h, and finally freeze-dried to obtain CBSA. BSA and CBSA were characterized by isoelectric focusing (IEF).

Characterization of core-shell nanocarriers.

Morphology. The morphologies of the BNCs, DNCs and CNCs were investigated using a transmission electron microscope (TEM). The nanocarrier samples were diluted appropriately in distilled water, and one drop was placed on a carbon-coated copper grid, followed by air drying and staining with 2% (w/v) phosphotungstic acid for 30 s at room temperature for contrast enhancement. Finally, the sample was observed by TEM (JEM-1230, Tokyo, Japan) after air drying.

Particle size, size distribution and zeta potential. The particle size, size distribution and zeta potential of the BNCs, DNCs and CNCs were measured using a Zetasizer 3000 HSA (Malvern Instruments Ltd., UK) according to the dynamic light scattering (DLS) principle. The samples were diluted appropriately in distilled water before measurement. Determinations were performed at 633 nm with a constant scattering angle of 90 ° at 25 ℃. The size distribution was indicated by the polydispersity index (PDI), and the raw data were expressed by the intensity-weighted Gaussian distribution (with Chi-squared value < 3). Each batch was carried out in triplicate.

Core-shell structure confirmation. The core-shell structure of nanocarriers was confirmed by confocal laser scanning microscopy (CLSM). Dual fluorescence-labeled microscale carriers labeled

with C6 and rhodamine B isothiocyanate (RBITC) were prepared using a procedure similar to the fabrication of BNCs. Briefly, the oil phase containing C6 (0.3 mg), EP (100 mg) and Labrafil® M 1944 CS (1 mL) was added to the aqueous phase containing 30 mL of 1.0% (w/v) RBITC-labeled CBSA and followed by dispersing at 10 000 rpm for 60 s using a high-speed disperser as described above. The suspension was diluted appropriately in distilled water and one drop of the sample was placed on the surface of a glass slide. After covering with a cover slip, the sample was observed by CLSM (Zeiss LSM 700, Germany).

RNA binding ability and protection assay. The RNA binding ability and protection of CBSA in the core-shell nanocarriers was investigated by agarose gel electrophoresis[2,3]. Briefly, CNCs were prepared at various weight ratios of CBSA/RNA (0.125, 1, 8, 32, 64, 128, 200 and 400). The samples were mixed with 5× loading buffer containing GelRed (Generay Biotechnology, China) and then electrophoresed on 2% (w/v) agarose gel in TBE buffer at 110 V for 10 min. Subsequently, the gel was photographed using chemiluminescence imaging system (ChemiDocTM XRS+, Bio-Rad, USA).

To evaluate the protection for miRNA-34a against RNase A degradation by CNCs, free miRNA-34a (0.5 μg) and CNCs (containing 0.5 μg miRNA-34a) were incubated with RNase A (final concentration 10 μg/mL) at 37 ℃ for the indicated times. The resulting samples were terminated by incubation with SDS (final concentration 1%) for 5 min at 60 ℃, and then heparin (final concentration 2%) was added to the samples to displace miRNAs from CNCs. Finally, the samples were analyzed by agarose gel electrophoresis as described above.

To assess the serum stability of miRNA-34a in CNCs, free miRNA-34a (0.5 μg) and CNCs (containing 0.5 μg miRNA-34a) were incubated in 10% FBS solution (final concentration) at 37 ℃ for the indicated periods. Then, the samples were picked out, processed according to the procedure described above and finally analyzed by agarose gel electrophoresis.

Differential scanning calorimetry (DSC) and powder X-ray diffraction (PXRD). To confirm the physical state of the DTX in the nanocarriers, DSC was performed using DSC (Q2000, TA Instruments, USA). The samples of pure DTX, CBSA, their physical mixture (PM), trehalose (protectant during freezing) and freeze-dried DNCs were sealed in aluminum pans and heated from

25-300 ℃ at a rate of 10 ℃/min under dry nitrogen atmosphere with a flow rate of 20 mL/min.

PXRD analysis was carried out using an X-ray powder diffractometer (D8 Advance, Bruker AXS, Germany). The X-ray tube was operated at 40 kV and 40 mA with Cu Kα radiation (λ =1.54060 Å) at room temperature. The scans over a range of 3 °- 40° (2θ) were obtained with a step of 0.02 °and step time of 0.3 s.

***In vitro* drug release.** The release profiles of DTX from DNCs and CNCs were carried out using a dialysis bag diffusion method. Briefly, 2 mL of the nanocarrier suspension (0.2 mg of DTX) was dialyzed in 30 mL of release medium (PBS containing 0.5% Tween 80, pH 7.4) using a dialysis bag with a molecular weight cutoff of 8-12 kDa. It was placed in a horizontal shaking incubator (SHA-C, Jintan, China) at 37 ℃ and 100 rpm of shaking speed. At designated time points, aliquots of release medium were taken out from the dialysate and replaced by an equal volume of release buffer. The DTX release from Duopafei® was measured in the same manner as a control. The concentration of DTX was determined by HPLC (LC-10ADvp pump, SPD10Avp UV-vis detector, Shimadzu Corporation, Tokyo, Japan). The chromatographic conditions were set as follows: Hypersil ODS column (4.6 mm × 250 mm, 5 μm); mobile phase: acetonitrile-water (55:45, V/V); detection wavelength: 230 nm; injection volume: 20 μL; flow rate: 1.0 mL/min; column temperature: 25 ℃.

***In vitro* leakage of C6 and Cy5-RNA from CNCs.** Briefly, 1 mL of dual fluorescence-labeled CNCs suspension were placed in two dialysis tubes with a molecular weight cutoff of 12 kDa for C6 and 50 kDa for Cy5-RNA, respectively. Then, the dialysis tubes were located in 10 mL of serum-free medium at 37 ℃ and under moderate shaking using a horizontal shaking incubator (SHA-C, Jintan, China). At indicated time intervals, 0.3 mL of release medium were taken out from the dialysate and replaced by an equal volume of release medium. In addition, C6 release from ^{C6}CNCs and Cy5-RNA release from CNCs$_{Cy5\text{-}RNA}$ were also measured in the same method. The concentration of C6 and Cy5-RNA were detected using a hybrid multi-mode microplate reader (POLARstar Omega, BMG Labtech, Germany). The excitation wavelength and the emission wavelength were set at 488 nm and 520 nm for C6, 630 nm and 670 nm for Cy5-RNA, respectively. Each sample was carried out in triplicate.

Caveolae-mediated cellular internalization and intracellular trafficking. To further verify the role of caveolae in the cellular uptake of CNCs, CTB-Alexa Fluor® 488 (or 594), the pathway marker of caveolae-mediated endocytosis, was used[4]. Briefly, 4T1 or Caco-2 cells were seeded in glass bottom dishes (35 mm) at a density of 1×10^5 cells/well and incubated for 24 h to allow cell attachment. To suppress caveolae-related internalization, one group of cells was pretreated with nystatin (20 μM) for 30 min at 37 °C. Then, the cells were incubated with serum-free medium containing fluorescence-labeled CNCs and CTB-Alexa Fluor® 488 (or 594) for 4 h at 37 ℃, following by washing with PBS three times, fixing with 4% formaldehyde for 10 min at room temperature, washing with PBS three times and finally visualization by CLSM.

To investigate the colocalization of CNCs with caveolae, anti-caveolin-1 antibody-Caveolae Marker (Alexa Fluors® 488 or 594) was used for caveolae staining[5]. Briefly, 4T1 or Caco-2 cells were seeded in a confocal special dish (35 mm) at a density of 1×10^5 cells/well and cultured for 24 h. One group of cells was pretreated with nystatin (20 μM) for 30 min at 37 °C. Then, the cells were incubated with serum-free medium containing fluorescence-labeled CNCs for 4 h at 37 ℃. After removing the media, washing with PBS three times, fixing with 4% formaldehyde for 10 min, permeabilization in 0.1% Triton X-100 for 5 min, and blocking with 5% BSA for 20 min at room temperature, the cells were incubated with anti-caveolin-1 antibody (Alexa Fluor® 488 or 594) overnight at 4 ℃ in the dark. The cells were observed by CLSM after washing with PBS.

To study the locations of the CNCs in the lysosomes, the distribution of the CNCs in living cells was examined by CLSM. Briefly, after incubation with fluorescence-labeled CNCs for 4 h at 37 ℃, removal of the media, and washing with PBS three times, the 4T1 cells were incubated with LysoTracker for 2 h at 37 °C according to the manufacturer’s instructions, followed by removal of the media, PBS washing and observing by CLSM.

Tumor spheroids penetration. To investigate the penetration of CNCs in tumors, 4T1 tumor spheroids were established according to a previously reported method[6]. Briefly, the 96-well plates were covered with 100 μL 2% agarose to prevent cell adhesion. Then, 4T1 cells were seeded at a density of 1×10^4 cells/well and cultures at 37 ℃ for spheroids formation. After incubation for about a week, the spheroids were treated with serum-free culture media containing dual

fluorescence-labeled CNCs for 4 h at 37 °C. Finally, the tumor spheroids were washed with fresh PBS three times and observed by CLSM.

***In vitro* RNAi experiments.** To assess the expression level of miRNA-34a, quantitative real-time polymerase chain reaction (qRT-PCR) was performed. Briefly, 4T1 cells were seeded in 6-well plates at a density of 3×10^5 cells/well and cultured for 24 h at 37 °C. Then, the cells were transfected with DTX, BNCs, DNCs, RNCs and CNCs, respectively, for 4 h with a fixed miRNA-34a concentration of 100 nM, following by medium removing and additional incubation with fresh medium for 48 h. The total RNA was extracted from the cultured cells using Trizol reagents (Invitrogen, USA) according to the manufacturer’s protocol. cDNA was obtained by reverse transcription using RevertAid First Strand cDNA Synthesis Kit (Thermo Scientific, USA). QRT-PCR was performed using an ABI Step One Plus Real-time PCR system (Applied Biosystems, USA) with SYBR® Green Real-time PCR Master Mix (TOYOBO, Japan). Twenty microliters of the reverse transcription mixture was amplified by PCR with the following program: 40 cycles at 95 °C for 15 s, 60 °C for 20 s, 72 °C for 40 s and 1 cycle at 95 °C for 15 s, 60 °C for 60 s and 95 °C for 15 s. GAPDH was used as internal reference for normalization.

For western-blot analysis, cultured cells were lysed in lysis buffer (KeyGEN, China) by incubation on ice for 20 min. Protein was collected by the following procedure: centrifuging the cells at 12 000 rpm for 10 min, collecting supernatants, determining concentration using BCA protein assay kit (KeyGEN Biotech., China) and Varioskan spectrophotometer (Thermo, USA), and separating by SDS-PAGE. The isolated protein was then transferred onto a PVDF membrane (Millipore, USA), incubated with the blocking solution (5% non-fat dried milk) at room temperature for 1 h, treated with monoclonal antibody against Bcl-2 overnight at 4 °C, and cultured with the goat anti-mouse secondary antibody for 1 h. After staining with an ECL chemiluminescence kit (KeyGEN Biotech., China), blotting images were obtained from a G: Box Chemi XR5 (Syngene, Cambridge, UK). GAPDH was used as an internal control to normalize the protein expression.

Cell cytotoxicity and apoptosis assays. Cell cytotoxicity was assessed by the MTT method. Briefly, 4T1 cells were seeded with a density of 5×10^3 cells/well in 96-well plates and cultured for

24 h for cell attachment. Then, the cells were exposed to fresh culture medium containing BNCs, DTX, DNCs, RNCs and CNCs, respectively. The concentration of miRNA-34a was fixed at 100 nM and the concentrations of DTX were set as 0.01, 0.1, 1, 10 and 50 μg/mL. After incubation for 48 h, 20 μL MTT (5 mg/mL) was added to each well, and the cells were incubated for an additional 4 h. The medium was removed, and then, 200 μL of dimethyl sulfoxide (DMSO) was added to each well to dissolve formazan crystals. Finally, the absorbance at 570 nm was measured using a Microplate Reader (Multiskan FC, USA). The cell viability was calculated by the following formula: Cell viability (%) = $(A_{sample}-A_{blank})/(A_{control}-A_{blank}) \times 100\%$, where A_{sample}, A_{blank}, and $A_{control}$ were the absorbance of the cells treated with the formulations, the culture media without the cells, and the cells without treatment.

Cell apoptosis was evaluated by the cell nuclear morphology after DAPI staining and AnnexinV-FITC/PI apoptosis detection kits, respectively. 4T1 cells were treated with fresh culture media containing DTX, BNCs, DNCs, RNCs and CNCs (10 μg/mL of DTX or 100 nM of miRNA-34a) for 48 h, respectively. The cells were washed three times with PBS, stained with DAPI at room temperature for 10 min, and observed by an inverted fluorescence microscope (Olympus IX53, Japan). Cell apoptosis was evaluated according to the nuclear morphology changes, such as chromatin condensation, fragmentation and apoptotic body formation. For quantitative analysis of cell apoptosis, AnnexinV-FITC/PI apoptosis detection kits and FCM were employed for determination. Cells were treated as described above, harvested after incubation with drug formulations, processed according to the manufacturer's protocol, and finally determined by FCM.

***In vitro* transwell migration assay.** The migration assay was performed using transwell chambers with a pore size of 8 μm (Corning, USA)[7]. Briefly, 4T1 cells were seeded in 6-well plates at a density of 5×10^5 cells/well and cultured for 24 h. Then, the cells were treated with serum-free culture media containing with DTX, BNCs, DNCs, RNCs and CNCs (10 μg/mL of DTX or 100 nM of miRNA-34a) for 24 h, respectively. After washing with PBS, harvesting and suspending in serum-free medium, 200 μL of the cell suspension was placed into the top chamber at a density of 1×10^5 cells/chamber, and 500 μL of RPMI 1640 medium containing 20% FBS as a chemoattractant was added to the lower chamber. After incubation for 24 h at 37 ℃, the cells in the upper chambers

were scraped off with a cotton swab; the migrated cells on the lower surface were fixed with 70% ethanol for 30 min, stained with 0.25% crystal violet for 15 min at room temperature, and washed with fresh PBS three times. Cell migration images were obtained using an inverted fluorescence microscope (Olympus IX53, Japan). Thereafter, the transwell inserts were incubated in 33% acetic acid to dissolve the crystal violet completely. To quantitatively detect the amounts of migrating cells, the absorbance (OD values) at 600 nm was measured using a Microplate Reader (Multiskan FC, USA). The cell migration rate was calculated by the following formula: Cell migration rate (%) = $OD_{sample}/OD_{control} \times 100\%$, where OD_{sample} and $OD_{control}$ were the absorbance of the migrating cells with treatment of different formulations and the migrating cells without treatment, respectively.

Pharmacokinetics in rats. The animals used in the experiments received care in compliance with the Principles of Laboratory Animal Care and the Guide for the Care and Use of Laboratory Animals. All the animal experiments were performed in accordance with the protocol approved by the China Pharmaceutical University Institutional Animal Care and Use Committee.

SD rats were randomly divided into two groups (n = 3) and then injected via the tail vein with Duopafei® and DNCs at a dose of 10 mg/kg DTX, respectively. Approximately 0.5 mL of blood samples were collected from rat orbit at predetermined time intervals (0.083, 0.25, 0.5, 1, 2, 4, 6, 8, 10 and 12 h), and plasma samples were obtained after centrifugation, extraction with ethyl ether and redissolution with methanol. The concentration of DTX was measured by HPLC. The pharmacokinetic parameters were calculated by Debris Assessment Software version 2.0 (DAS 2.0, NASA Orbital Debris Program Office, Houston, TX).

***In vivo* imaging and biodistribution.** 4T1 tumor-bearing mice models were established by subcutaneous injection of 0.1 mL PBS containing 1×10^6 4T1 cells at the right axilla of BALB/c mice. The mice were used in the experiments when the tumor volume reached approximately 500 mm^3. Dual fluorescence-labeled CNCs (loaded with C6 and Cy5-RNA) were injected via the tail vein at a C6 dose of 2 mg/kg and Cy5-RNA dose of 1 mg/kg with free C6 and free Cy5-RNA as controls[8-10]. The *in vivo* fluorescence signals were detected using an *in vivo* imaging system (FX PRO, Kodak, USA) at predetermined intervals (1 h, 4 h, 8 h and 24 h). To detect the fluorescence intensity, the bandpass filter was fixed at excitation wavelengths of 470 nm and 630 nm and

emission wavelengths of 530 nm and 670 nm for C6 and Cy5, respectively. To further investigate the biodistribution of the CNCs, the mice were sacrificed at the indicated time after administration to collect the tumor, heart, liver, spleen, lung and kidney for imaging.

To further confirm the distribution of the CNCs in the tumors, the tumor tissues were excised from the mice at 4 h post-injection of dual fluorescence-labeled CNCs. The sample for CLSM observation was prepared as follows: freeze the excised tumor tissue, cut into sections (6-8 μm) using a microtome (Leica CM1860, Germany) and stain with DAPI. For the TEM examination, the procedure for sample preparation was followed by cutting the isolated tumor tissue into small blocks with a volume of approximately 1 mm3, fixing in 2.5% glutaraldehyde overnight, dehydration, penetration, embedding, preparing thin sections (approximately 50 nm) and staining with uranyl acetate and lead citrate.

***In vivo* safety study.** Normal BALB/c mice were randomly divided into six groups (n = 5) and injected with different formulations (saline, Duopafei®, BNCs, DNCs, RNCs and CNCs) by the same administration schedule as described in the *in vivo* antitumor experiments. The mice were sacrificed after administration, and the main organs including hearts, lives, spleens, lungs and kidneys were harvested for H&E and CD68 immunohistochemical analysis.

References

1 Lu, W. *et al.* Cationic albumin-conjugated pegylated nanoparticles as novel drug carrier for brain delivery. *J Control Release* **107**, 428-448 (2005).

2 Jiang, Y. *et al.* Direct cytosolic delivery of siRNA using nanoparticle-stabilized nanocapsules. *Angew Chem Int Ed Engl* **54**, 506-510 (2015).

3 Han, J., Wang, Q., Zhang, Z., Gong, T. & Sun, X. Cationic bovine serum albumin based self-assembled nanoparticles as siRNA delivery vector for treating lung metastatic cancer. *Small* **10**, 524-535 (2014).

4 Song, Q. *et al.* Cellular internalization pathway and transcellular transport of pegylated polyester nanoparticles in Caco-2 cells. *Int J Pharm* **445**, 58-68 (2013).

5 Tiruppathi, C. *et al.* Albumin mediates the transcytosis of myeloperoxidase by means of caveolae in endothelial cells. *Proc Natl Acad Sci U S A* **101**, 7699-7704 (2004).

6 Zhu, L., Wang, T., Perche, F., Taigind, A. & Torchilin, V. P. Enhanced anticancer activity of nanopreparation containing an MMP2-sensitive PEG-drug conjugate and cell-penetrating moiety. *Proc Natl Acad Sci U S A* **110**, 17047-17052 (2013).

7 Zheng, Y. *et al.* CA1 contributes to microcalcification and tumourigenesis in breast cancer. *BMC Cancer* **15**, 679 (2015).

8 Yin, T., Wang, L., Yin, L., Zhou, J. & Huo, M. Co-delivery of hydrophobic paclitaxel and hydrophilic AURKA specific siRNA by redox-sensitive micelles for effective treatment of breast cancer. *Biomaterials* **61**, 10-25 (2015).

9 Shi, S. *et al.* Dual drugs (microRNA-34a and paclitaxel)-loaded functional solid lipid nanoparticles for synergistic cancer cell suppression. *J Control Release* **194**, 228-237 (2014).

10 Tang, S. *et al.* Tumor‐Microenvironment‐Adaptive Nanoparticles Codeliver Paclitaxel and siRNA to Inhibit Growth and Lung Metastasis of Breast Cancer. *Adv Funct Mater* (2016).

11 Naik, S., Patel, D., Surti, N. & Misra, A. Preparation of PEGylated liposomes of docetaxel using supercritical fluid technology. *J Supercrit Fluid* **54**, 110-119 (2010).

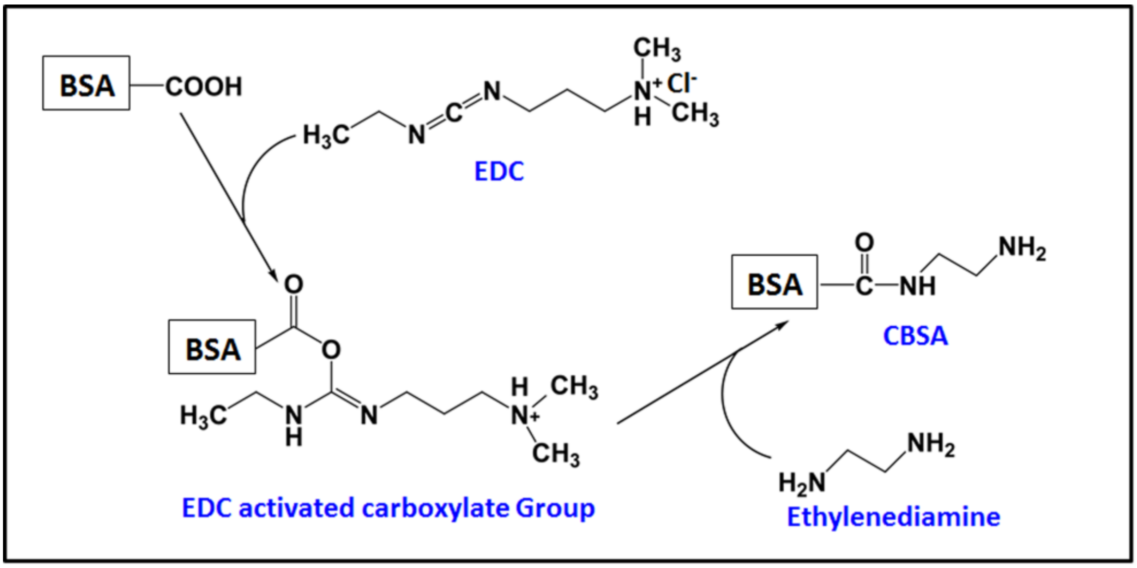

Fig. S1 Synthesis scheme of CBSA.

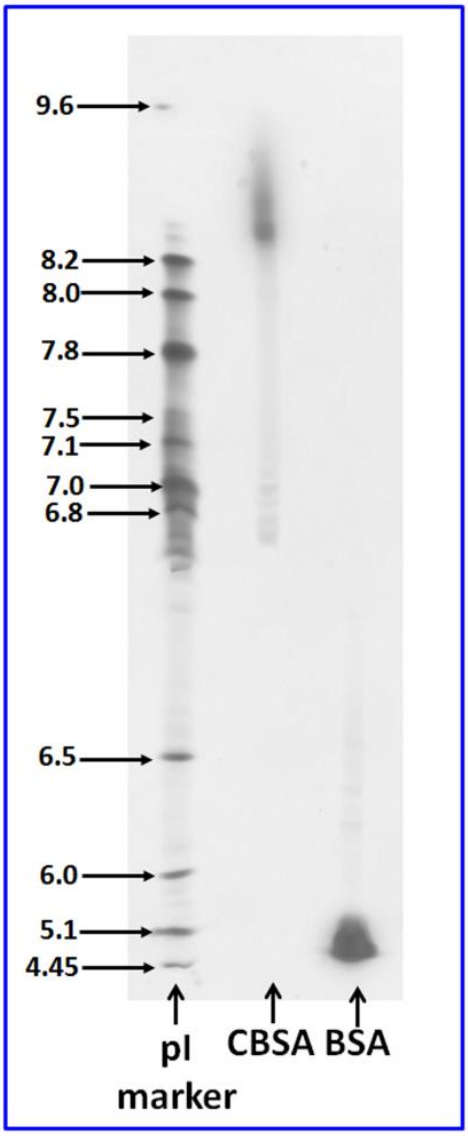

Fig. S2 IEF diagram of BSA and CBSA. The pI values of BSA and CBSA were 4.6 and 8-9, respectively.

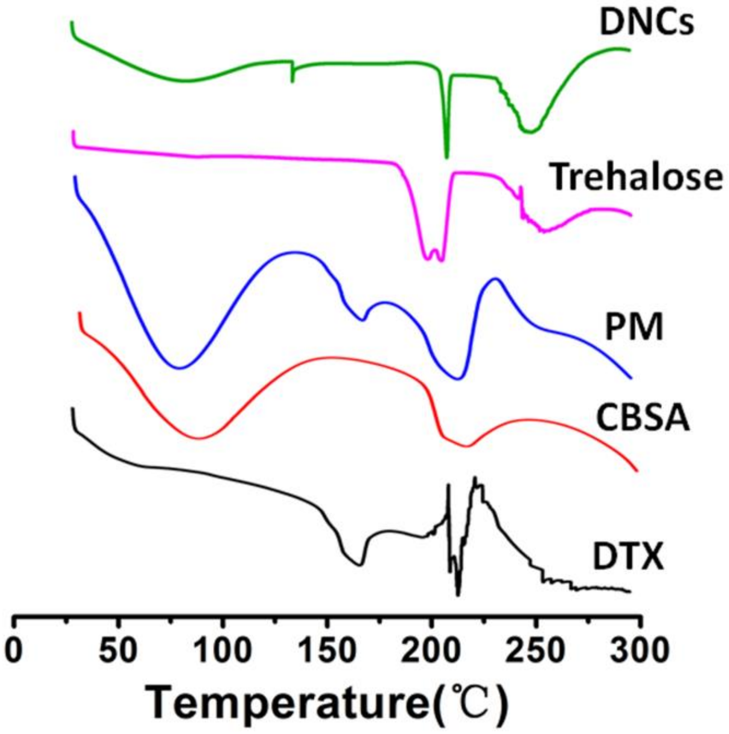

Fig. S3 DSC analysis of pure DTX, CBSA, their physical mixture (PM), trehalose and freeze-dried DNCs. DTX showed two strong endothermic melting peaks at 167.9 ℃ and 211.4 ℃, which is consistent with the previously reported results.[11] CBSA was amorphous macromolecular protein; thus, it showed two broad peaks. The PM sample also possessed the two characteristic diffraction peaks of DTX. Trehalose, the protectant during freezing, showed melting peaks from 200 ℃ to 206 ℃. For the DSC thermograms of freeze-dried DNCs, the characteristic peak of DTX at 167.9 ℃ was absent, indicating that DTX in nanocarriers was in the amorphous phase of a molecular dispersion and thus was well encapsulated in the cores of DNCs.

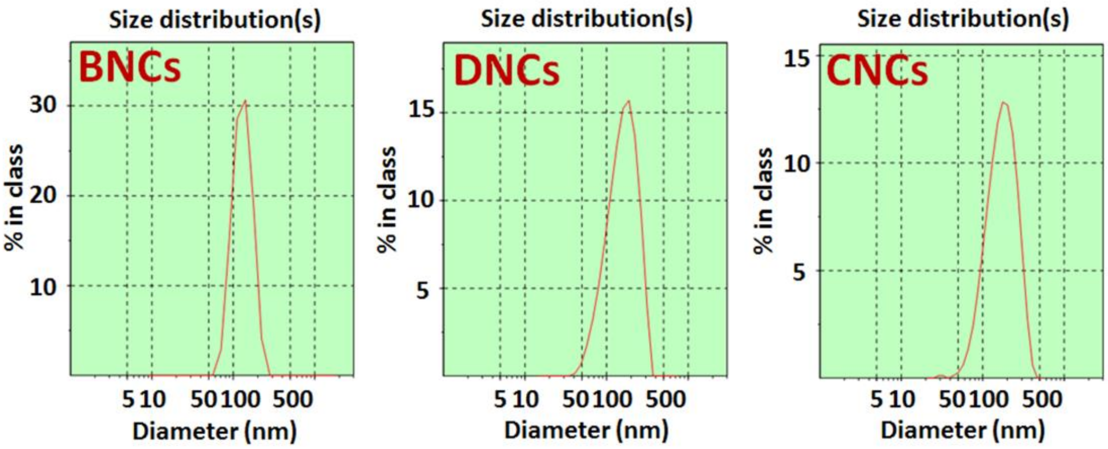

Fig. S4 Size distributions of the BNCs, DNCs and CNCs determined by dynamic light scattering (DLS).

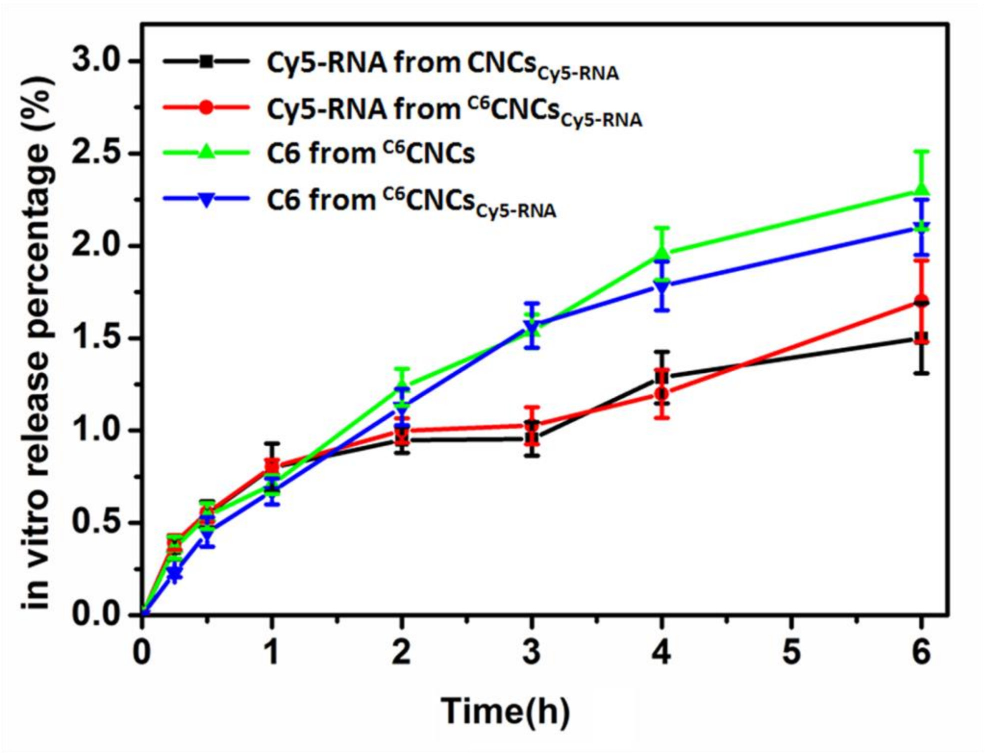

Fig. S5 *In vitro* leakage of C6 and Cy5-RNA from CNCs in serum-free medium at 37 ℃. ^{C6}CNCs: C6-loaded CNCs; CNCs$_{Cy5-RNA}$: Cy5-RNA loaded CNCs; ^{C6}CNCs$_{Cy5-RNA}$: C6 and Cy5-RNA dual fluorescence-labeled CNCs.

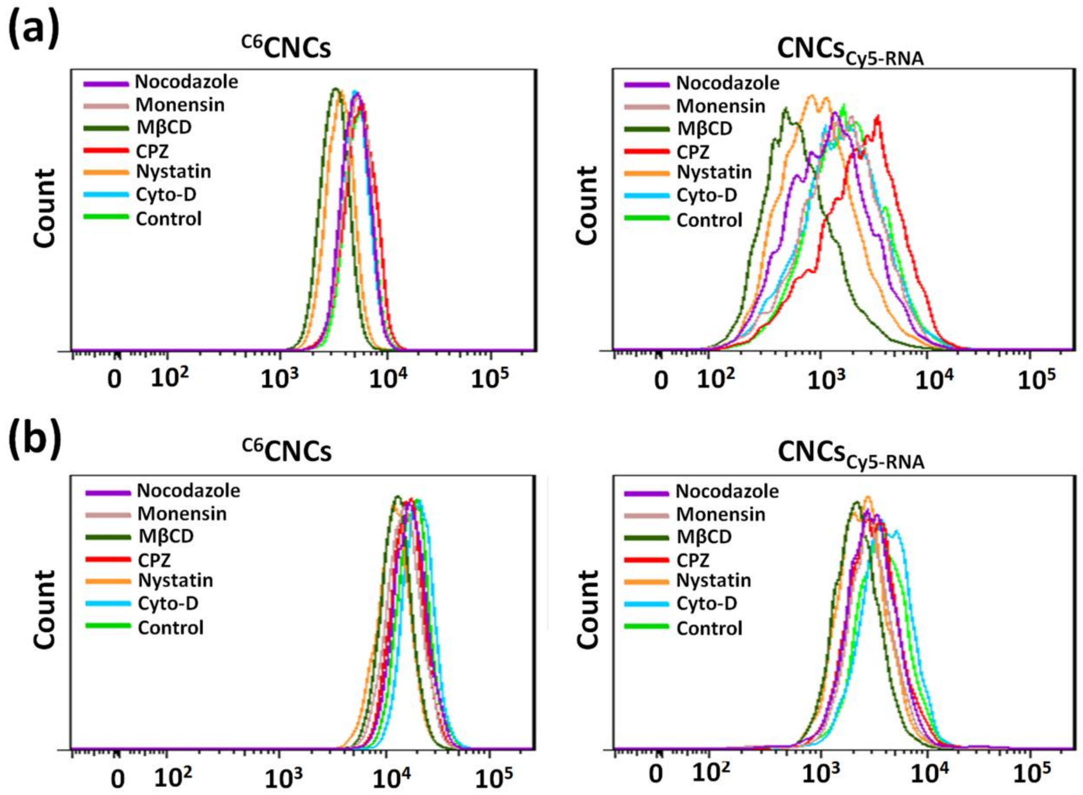

Fig. S6 FCM results of the cell uptake of CNCs in (a) 4T1 cells and (b) Caco-2 cells pretreated with various endocytosis inhibitors.

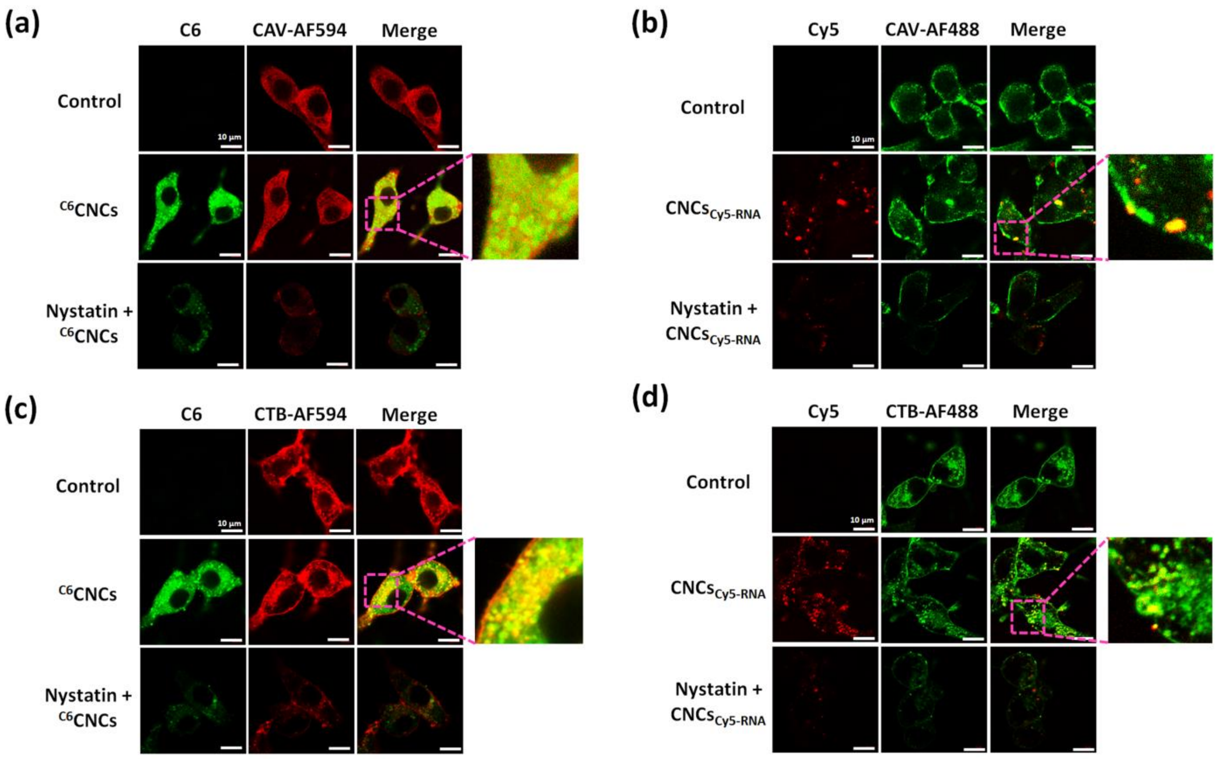

Fig. S7 Caveolae-mediated cellular internalization of CNCs in Caco-2 cells. Colocalization of (a) ^{C6}CNCs or (b) CNCs$_{Cy5\text{-}RNA}$ with Caveolin-1 (CAV-1) after incubation for 4 h at 37 ℃ in the absence or presence of nystatin. CNCs were labeled by C6 (green) or Cy5-RNA (red). CAV-1 was marked by anti-caveolin-1 antibody/Alexa Fluors® 594 (red) or 488 (green). Colocalization of (c) ^{C6}CNCs or (d) CNCs$_{Cy5\text{-}RNA}$ with CTB-Alexa Fluors® 594 (red) or 488 (green) after incubation for 4 h at 37 ℃ in the absence or presence of nystatin. Yellow spots indicate the colocalization of CNCs with caveolae or CTB. Caco-2 cells without treatment were used as control. Scale bar: 10 μm

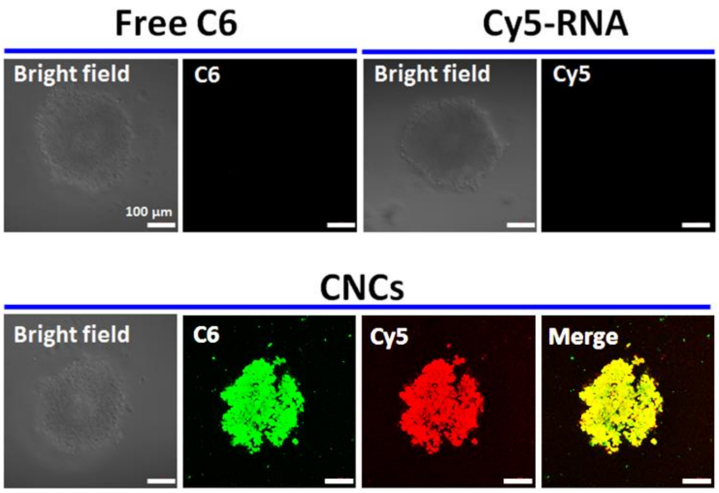

Fig. S8 *In vitro* penetration of CNCs in 4T1 tumor spheroids after incubation for 4 h at 37 ℃. CLSM images were obtained from the equatorial plane of the tumor spheroids. Scale bar: 100 μm.

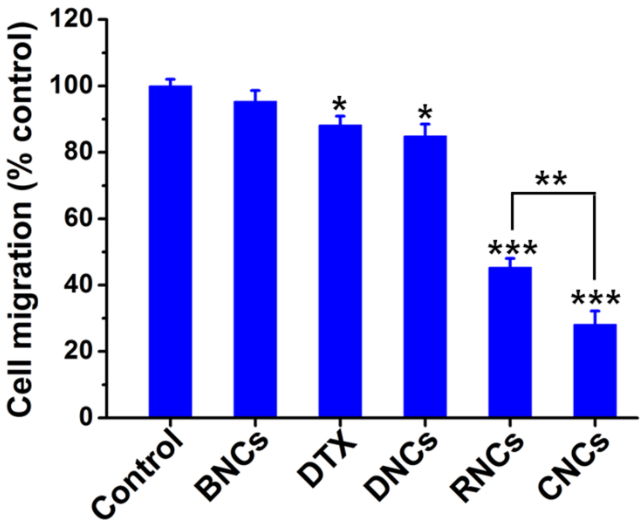

Fig. S9 Quantitative analysis of the inhibition migration of 4T1 cells examined by transwell chamber assay. The migrated cells were represented by the absorbance of crystal violet measured at 600 nm. The cell migration of the control group was set as 100%. $^{*}P < 0.05$, $^{**}P < 0.01$, $^{***}P < 0.001$ versus control.

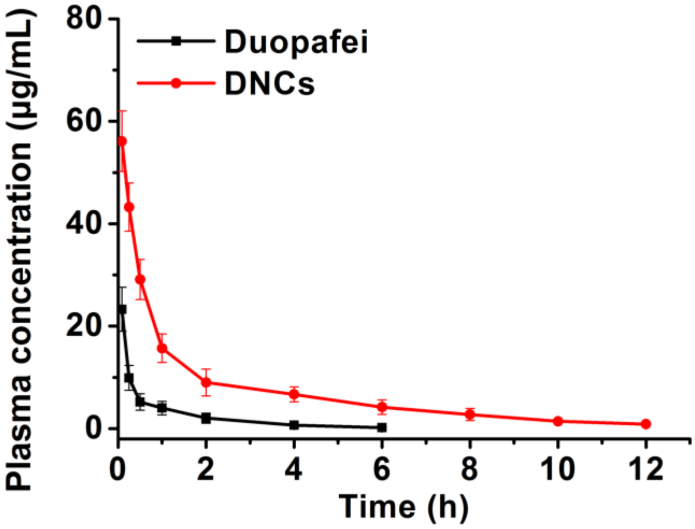

Fig. S10 The mean plasma concentration–time curves of DTX after intravenous injection of Duopafei® and DNCs at a dose of 10 mg/kg in SD rats (n = 3).

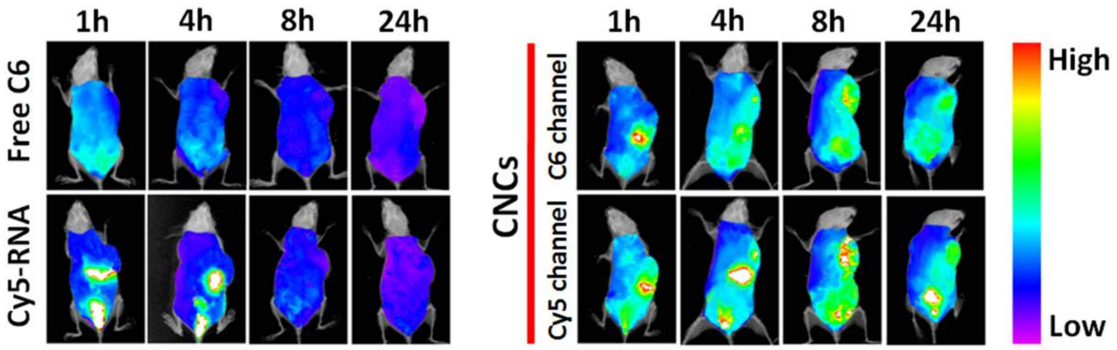

Fig. S11 *In vivo* imaging of free C6, Cy5-RNA and dual fluorescence-labeled CNCs in 4T1-tumor bearing mice models. Dual fluorescence-labeled CNCs were prepared by loading C6 (green) in the cores and Cy5-RNA (red) in the shells of CNCs.

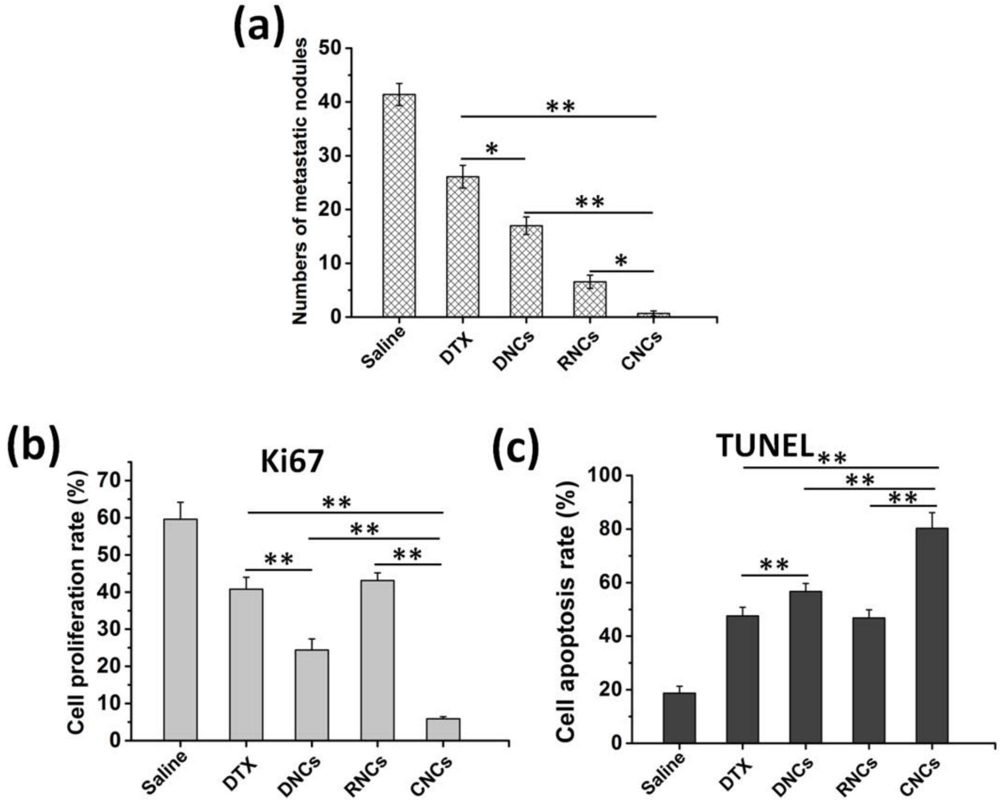

Fig. S12 (a) Quantitative analysis of the metastatic nodules in lungs. (b) Quantitative analysis of cell proliferation from Ki67 assays. (c) Quantitative analysis of cell apoptosis from TUNEL assays. $^{*}P<0.05$, $^{**}P<0.01$, $^{***}P<0.001$.

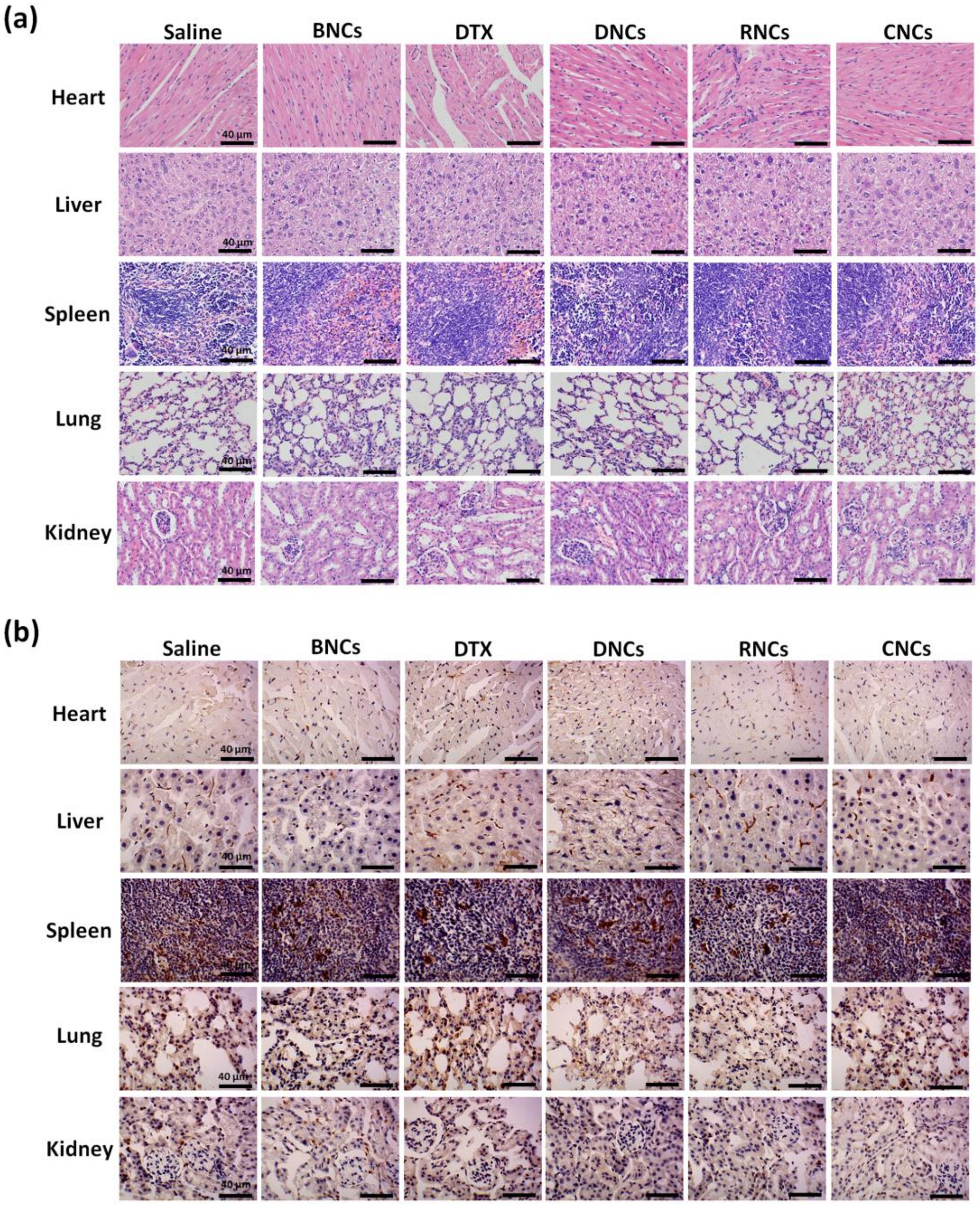

Fig. S13 *In vivo* safety studies. (a) Histological examination of major organs harvested from normal mice after treatment with saline, BNCs, DTX, DNCs, RNCs and CNCs. Scale bar: 40 μm. (b) CD68 immunohistochemical assay of major organs harvested from normal mice after treatment with saline, BNCs, DTX, DNCs, RNCs and CNCs (400 ×). Scale bar: 40 μm.

Table S1 Particle size, polydispersity index (PDI) and zeta potential of the BNCs, DNCs and CNCs.

	**BNCs**	**DNCs**	**CNCs**
Size (nm)	155.3 ±3.5	171.1 ±5.3	183.9 ±2.8
PDI	0.176 ±0.034	0.191 ±0.017	0.195 ±0.012
Zeta potential (mV)	28.87 ±4.94	28.49 ±3.97	23.37 ±3.89

Table S2 Pharmacokinetic parameters of DTX after intravenous injection of Duopafei® and DNCs at a dose of 10 mg/kg in SD rats (n = 3).

**Pharmacokinetic parameters**	**Formulations**	
	**Duopafei®**	**DNCs**
$t_{1/2}$ (h)	1.17 ± 0.14	3.52 ± 0.26
$AUC_{0-\infty}$ (mg·h/L)	15.99 ± 2.31	93.15 ± 5.63
$MRT_{0-\infty}$ (h)	1.25 ± 0.34	3.423 ± 0.29
CL (L/h/kg)	0.625 ± 0.073	0.107 ± 0.044
